# Genome-wide association scan identifies new variants associated with a cognitive predictor of dyslexia

**DOI:** 10.1038/s41398-019-0402-0

**Published:** 2019-02-11

**Authors:** Alessandro Gialluisi, Till F. M. Andlauer, Nazanin Mirza-Schreiber, Kristina Moll, Jessica Becker, Per Hoffmann, Kerstin U. Ludwig, Darina Czamara, Beate St Pourcain, William Brandler, Ferenc Honbolygó, Dénes Tóth, Valéria Csépe, Guillaume Huguet, Andrew P. Morris, Jacqueline Hulslander, Erik G. Willcutt, John C. DeFries, Richard K. Olson, Shelley D. Smith, Bruce F. Pennington, Anniek Vaessen, Urs Maurer, Heikki Lyytinen, Myriam Peyrard-Janvid, Paavo H. T. Leppänen, Daniel Brandeis, Milene Bonte, John F. Stein, Joel B. Talcott, Fabien Fauchereau, Arndt Wilcke, Clyde Francks, Thomas Bourgeron, Anthony P. Monaco, Franck Ramus, Karin Landerl, Juha Kere, Thomas S. Scerri, Silvia Paracchini, Simon E. Fisher, Johannes Schumacher, Markus M. Nöthen, Bertram Müller-Myhsok, Gerd Schulte-Körne

**Affiliations:** 1 Department of Translational Research in Psychiatry, Max Planck Institute of Psychiatry, Munich, Germany; 2 Munich Cluster for Systems Neurology (Sypartially), Munich, Germany; 3 Department of Epidemiology and Prevention, IRCCS Istituto Neurologico Mediterraneo Neuromed, Pozzilli, Italy; 4 Department of Child and Adolescent Psychiatry, Psychosomatic, and Psychotherapy, Ludwig-Maximilians University, Munich, Germany; 5 Institute of Human Genetics, University of Bonn, Bonn, Germany; 6 Department of Genomics, Life & Brain Center, University of Bonn, Bonn, Germany; 7 Language and Genetics Department, Max Planck Institute for Psycholinguistics, Nijmegen, Netherlands; 8 Donders Institute for Brain, Cognition and Behaviour, Radboud University, Nijmegen, Netherlands; 9 MRC Integrative Epidemiology Unit, University of Bristol, Bristol, UK; 10 University of California San Diego, Department of Psychiatry, San Diego, CA USA; 11 Brain Imaging Centre, Research Centre of Natural Sciences of the Hungarian Academy of Sciences, Budapest, Hungary; 12 Human Genetics and Cognitive Functions Unit, Institut Pasteur, Paris, France; 13 University Paris Diderot, Sorbonne Paris Cité, Paris, France; 14 Department of Biostatistics, Universiy of Liverpool, Liverpool, UK; 15 Wellcome Trust Centre for Human Genetics, University of Oxford, Oxford, UK; 16 Institute for Behavioral Genetics and Department of Psychology and Neuroscience, University of Colorado Boulder, Boulder, CO USA; 17 Developmental Neuroscience Munroe-Meyer Institute, University of Nebraska Medical Center, Omaha, NE USA; 18 Developmental Neuropsychology Lab & Clinic, Department of Psychology, University of Denver, Denver, CO USA; 19 Department of Cognitive Neuroscience, Faculty of Psychology and Neuroscience & Maastricht Brain Imaging Center (M-BIC), Maastricht University, Maastricht, Netherlands; 20 Department of Psychology, The Chinese University of Hong Kong, Shatin, N.T. Hong Kong; 21 Centre for Research on Learning and Teaching, Department of Psychology, University of Jyväskylä, Jyväskylä, Finland; 22 Department of Biosciences and Nutrition, Karolinska Institutet, Huddinge, Sweden; 23 Department of Child and Adolescent Psychiatry and Psychotherapy, Psychiatric Hospital, University of Zurich, Zurich, Switzerland; 24 Zurich Center for Integrative Human Physiology (ZIHP), Zurich, Switzerland; 25 Department of Child and Adolescent Psychiatry and Psychotherapy, Central Institute of Mental Health, Medical Faculty Mannheim/Heidelberg University, Mannheim, Germany; 26 Neuroscience Center Zurich, University of Zurich and ETH Zurich, Zurich, Switzerland; 27 Department of Physiology, University of Oxford, Oxford, UK; 28 School of Life and Health Sciences, Aston University, Birmingham, UK; 29 Cognitive Genetics Unit, Fraunhofer Institute for Cell Therapy and Immunology, Leipzig, Germany; 30 Tufts University, Medford, MA USA; 31 Laboratoire de Sciences Cognitives et Psycholinguistique, Ecole Normale Supérieure, CNRS, EHESS, PSL Research University, Paris, France; 32 Institute of Psychology, University of Graz, Graz, Austria and BioTechMed, Graz, Austria; 33 Molecular Medicine Program, Biomedicum, University of Helsinki, and Folkhälsan Institute of Genetics, Helsinki, Finland; 34 School of Basic and Medical Biosciences, King’s College London, London, UK; 35 The Walter and Eliza Hall Institute of Medical Research & Melbourne University, Melbourne, Australia; 36 School of Medicine, University of St Andrews, St Andrews, UK; 37 Institute of Translational Medicine, University of Liverpool, Liverpool, UK

## Abstract

Developmental dyslexia (DD) is one of the most prevalent learning disorders, with high impact on school and psychosocial development and high comorbidity with conditions like attention-deficit hyperactivity disorder (ADHD), depression, and anxiety. DD is characterized by deficits in different cognitive skills, including word reading, spelling, rapid naming, and phonology. To investigate the genetic basis of DD, we conducted a genome-wide association study (GWAS) of these skills within one of the largest studies available, including nine cohorts of reading-impaired and typically developing children of European ancestry (*N* = 2562–3468). We observed a genome-wide significant effect (*p* < 1 × 10^−8^) on rapid automatized naming of letters (RANlet) for variants on 18q12.2, within *MIR924HG* (*micro-RNA 924 host gene*; rs17663182 *p* = 4.73 × 10^−9^), and a suggestive association on 8q12.3 within *NKAIN3* (encoding a cation transporter; rs16928927, *p* = 2.25 × 10^−8^). rs17663182 (18q12.2) also showed genome-wide significant multivariate associations with RAN measures (*p* = 1.15 × 10^−8^) and with all the cognitive traits tested (*p* = 3.07 × 10^−8^), suggesting (relational) pleiotropic effects of this variant. A polygenic risk score (PRS) analysis revealed significant genetic overlaps of some of the DD-related traits with educational attainment (EDUyears) and ADHD. Reading and spelling abilities were positively associated with EDUyears (*p* ~ [10^−5^–10^−7^]) and negatively associated with ADHD PRS (*p* ~ [10^−8^−10^−17^]). This corroborates a long-standing hypothesis on the partly shared genetic etiology of DD and ADHD, at the genome-wide level. Our findings suggest new candidate DD susceptibility genes and provide new insights into the genetics of dyslexia and its comorbities.

## Introduction

Developmental dyslexia (DD) is a neurodevelopmental disorder affecting the ability of learning to read and to spell, in spite of adequate intelligence, educational opportunities, and in the absence of overt neurological and sensorial deficits^1^. It shows a prevalence of 5–12% among school-aged children, implying life-long learning difficulties for most of the affected individuals^1^. DD is characterized by a high rate of comorbidity with other neuropsychiatric conditions like attention-deficit hyperactivity disorder (ADHD), depression, and anxiety disorders^2^. Dyslexic individuals usually have severe and persistent problems in accurate and fluent reading and spelling, and in reading comprehension^3^. These problems are often associated with early deficits in neurocognitive skills, such as the ability to recognize and manipulate the phonemic constituents of speech (also known as phoneme awareness, PA), the ability to store such phonemes while reading (also known as phonological short-term memory), or the ability to fast map known visual symbols onto spoken word representations (known as naming speed)^4^. All these abilities show moderate-to-high heritability (40–80%)^5–7^ and significant genetic correlations with DD^5^. Hence, they represent cognitive indicators of dyslexia risk that are optimally suited for investigating the genetic mechanisms at its basis.

In the last two decades, several studies investigating both DD and the underlying cognitive skills have been carried out to better understand the genetic and neurobiological basis of dyslexia. On the one hand, linkage and targeted association analyses have suggested different candidate DD susceptibility genes (reviewed in refs. ^1,8,9^). Only a few of these genes have been implicated in DD, reading ability, and underlying cognitive skills in at least two independent studies or datasets^1^. These include *DYX1C1* (15q21)^10^, *KIAA0319* (6p22)^11–14^, *DCDC2* (6p22)^15–18^, *MRPL19/GCFC2* (2p12)^19^, *ROBO1* (3p12)^20–22^, *GRIN2B*^23,24^, *FOXP2*^25–27^ and *CNTNAP2*^27–29^.

On the other hand, most of the genome-wide association studies (GWAS) published so far have identified mainly suggestive associations with DD and related cognitive traits (*p* < 10^−5^)^30–34^, with only one recent study reporting a genome-wide significant association (*p* < 5 × 10^−8^; see below)^35^. The first GWAS for reading ability used DNA pooling of low vs. high reading ability groups in ~1500 7-year-old children, which were genotyped with a low-density single-nucleotide polymorphism (SNP) microarray (∼107,000 SNPs)^34^. The SNPs showing the largest allele frequency differences between low- and high-ability groups were tested in an additional follow-up cohort of 4258 children, finally identifying 10 SNPs showing nominally significant associations with continuous variation in reading ability^34^. However, Luciano et al.^36^ later found no evidence of replication of these findings in an adolescent population sample of Australian twins and siblings (*N* = 1177). A later genome-wide linkage and association scan on ∼133,000 SNPs, in a sample of 718 subjects from 101 dyslexia-affected families, identified an association with dyslexia status at rs9313548, near *FGF18* (5q35.1)^33^. More recently, three GWAS studies with different designs were carried out with the aim of identifying shared genetic contributions to reading and language abilities. Luciano et al.^32^ performed a GWAS on quantitative reading- and language-related traits in two population-based cohorts (*N* ∼ 6500), analyzing word reading, nonword repetition, and a composite score of reading and spelling abilities. They reported a suggestive association of rs2192161 (*ABCC13*; 21q11.2) with nonword repetition and of rs4807927 (*DAZAP1*, 19p13.3) with both the word reading and the reading–spelling score. A case-control GWAS comparing dyslexic (*N* = 353), language impaired (LI) (*N* = 163), and comorbid cases (*N* = 174) to a population-based control dataset (*N* = 4117) identified nominally significant associations with comorbid DD-LI cases drawn from the same population cohort used by Luciano et al.^32^. The most significant associations were detected at rs12636438 and rs1679255, mapping to *ZNF385D* (3p24.3)^30^. Another GWAS analyzed the first principal component from various reading- and language-related traits (both with and without IQ adjustment) in three datasets comprising children with reading or language problems and their siblings (*N* = 1862), and reported suggestive associations at rs59197085, upstream of *CCDC136/FLNC* (7q32.1), and at rs5995177, within *RBFOX2* (22q12.3)^31^. More recently, Truong et al.^35^ reported a genome-wide significant multivariate association of rs1555839 (10q23.31) with two skills predicting DD risk, namely rapid automatized naming (RAN) and rapid alternating stimulus (RAS), in a multisite case-control study of DD made up of individuals of non-European ancestry (*N* = 1263). This SNP, located upstream of the pseudogene *RPL7P34*, was also associated with measures of word reading and was replicated with RAN traits in an independent cohort from Colorado^35^, partially overlapping with the Colorado dataset analyzed by Gialluisi et al.^31^.

Although many of the genes suggested by these GWAS studies showed interesting potential biological links to DD and underlying skills, most of these associations did not reach genome-wide significance and were not replicated in independent datasets^36,37^. Prominently, an analysis of 17 candidate SNPs mostly identified by these GWAS, did not manage to replicate the associations previously reported, with word/nonword reading and fluency, PA and RAN traits, in an independent family-based Dutch population dataset comprising 483 children and 505 parents from 307 nuclear families^37^. This might have different reasons, including the low statistical power of the original GWAS studies implied by the relatively small sample sizes, and the heterogeneity of recruitment criteria and phenotypic assessment of the cohorts involved. In addition, the candidate susceptibility genes identified and replicated so far explain only a minor part of the genetic variance underlying dyslexia and related cognitive traits, and a big proportion of this heritability remains unexplained.

To help unravel the genetic basis of DD and related neurocognitive skills, we conducted a large international collaborative GWAS. We analyzed the cognitive traits word reading, spelling, decoding skills, phoneme awareness, verbal short-term memory, and naming speed, in nine cohorts of reading impaired and typically developing participants of European ancestry (maximum *N* = 3468). We observed a genome-wide significant association at 18q12.2 and an association approaching genome-wide significance at 8q12.3, both with rapid automatized naming (RAN, *N* = 2563). These genetic effects extended beyond the RAN domain, to other DD-related skills. Also, we detected significant genetic overlaps of the traits analyzed with educational attainment and ADHD risk. Overall, our findings provide new insights in the genetic etiology of dyslexia and related cognitive traits.

## Subjects and methods

### Datasets

Table 1 reports the main details on the datasets involved in this study and on the recruitment criteria.

**Table 1:** Main characteristics and recruitment criteria of the datasets involved in the present study

Dataset	Recruitment	Language	Relationships	Sex ratio (M:F)	Age range, years: (mean, SD)	IQ inclusion criteria
AGS^a^	DD cases and controls	German	Only unrelated subjects	886:568	8-19 (10.7, 2.4)	Age-appropriate WISC block design^114,115^ score ≥ 7; age-appropriate WISC similarities^114,115^ score ≥ 6
Finland	DD cases and controls	Finnish		167:157		
France	DD cases and controls	French		94:69		
Hungary	DD cases and controls	Hungarian		136:105		
Netherlands	DD cases and controls	Dutch		157:127		
Colorado	Children with a DD school history and their siblings	English	Siblings (small nuclear families)	292:258	8-19 (11.5, 2.7)	Full scale IQ (average score of age-adjusted WISC-R/WAIS-R verbal IQ and performance IQ, measured through multiple subtests)^116^ ≥ 80^c^
UK^b^	DD cases and their siblings	English		596:327	5-31 (11.8, 3.6)	Full scale IQ (average of age-adjusted standardized BAS/WAIS-R similarities subtest and BAS matrices subtest score)^117,118^ ≥ 80^c^

*IQ* Intelligence Quotient, *DD* developmental dyslexia, *WISC* Wechsler Intelligence Scale for Children, *WAIS* Wechsler Adult Intelligence Scale – Revised, *BAS* British Ability Scale

^a^Austria–Germany–Switzerland

^b^United Kingdom

^c^See ref. ^31^ for details

Unrelated DD cases and controls were recruited across seven different European countries, namely Austria (*N* = 374), Germany (*N* = 1061), Finland (*N* = 336), France (*N* = 165), Hungary (*N* = 243), The Netherlands (*N* = 311), and Switzerland (*N* = 67). Cases were defined as subjects showing more than 1.25 standard deviations (SD) below grade level on a standardized word reading test, while controls were defined as subjects with less than 0.85 SD below grade level on the same test^38^. In addition, we included two family-based datasets in the study. One of these, from Colorado, United States (USA), contained children showing a school history of reading difficulties as well as their siblings (*N* = 585; 266 independent nuclear families)^31,39^. The other one, from the United Kingdom (UK), consisted of subjects with a formal diagnosis of dyslexia and their unaffected siblings (*N* = 983; 608 independent nuclear families)^31,40^. Ethical approval was obtained for each cohort at the local level, and written informed consent was obtained for all the participants or their parents, as described elsewhere^31,41,42^.

Although the family-based datasets have been previously investigated in GWAS studies^31,40,43^, the European datasets have been analyzed in a candidate (SNP) association study^42^, and part of the German dataset has been investigated in relation to mathematical abilities^23^ and to neurophysiological DD endophenotypes^44^, such datasets were never analyzed jointly in a GWAS of neurocognitive traits related to dyslexia. In the present study, samples from Austria, Germany, and Switzerland were merged into a single dataset (hereafter called AGS), since they shared language, genetic ancestry, phenotypic measures, and selection criteria^38,42,45^. No other cohorts were approached for the present study, and all the cohorts contacted presented no refusal or lack of requirements for inclusion criteria.

### Phenotypic measures

We focused on the core phenotypes of dyslexia, namely word reading (WRead), nonword reading (NWRead), and word spelling (WSpell), and on five neurocognitive measures underlying reading ability and dyslexia (as well as other comorbid learning disabilities, e.g., dyscalculia). These skills included phoneme awareness (PA), digit span (DigSpan, a measure of verbal short-term memory), and rapid automatized naming of letters (RANlet), digits (RANdig), and pictures (RANpic). These traits showed moderate-to-high cross-trait correlations (see Table S1a, b in Supplementary Methods). A brief explanation of these measures is reported in Table 2, while details on statistical elaboration are reported in Supplementary Methods and elsewhere^31,38,45^. Briefly, raw scores from psychometric tests were grade-normed (age-adjusted in Colorado) and then z-standardized to reduce skewness, with the exception of the DigSpan score, which was only z-normalized^38,45^. No phenotypic outliers were detected in any of the datasets analyzed (see Supplementary Methods for details).

**Table 2:** Cognitive traits analyzed in the present study

Trait	Definition	Task
Wread	Reading single real words of varied difficulty	Timed word reading in AGS, Finland, France, Hungary, and the Netherlands; Untimed word reading in UK; composite score of timed word reading and reading accuracy in Colorado
Wspell	Spelling single real words after dictation	Spelling accuracy
NWRead	Reading aloud nonsense words of varied difficulty	Timed nonword reading in AGS, Finland, France, Hungary, and the Netherlands; untimed nonword reading in UK and Colorado
PA	Deletion, substitution or swapping of specific phonemes in one or multiple words	Phoneme deletion in AGS, Finland, France, Hungary, and the Netherlands; Phoneme deletion/substitution and spoonerism in UK; composite of phoneme deletion and phoneme segmentation and transposition tasks in Colorado
DigSpan	Reciting a sequence of digits presented by recalling them in the same (forward) and/or reverse (backward) order	WISC (Wechsler intelligence scale for children) forward and backward digit span task
RANdig	Naming as quickly and as accurately as possible a matrix of digits visually presented	Naming speed task (number of digits correctly named per minute)
RANlet	Naming as quickly and as accurately as possible a matrix of letters visually presented	Naming speed task (number of letters correctly named per minute)
RANpic	Naming as quickly and as accurately as possible a matrix of objects visually presented	Naming speed task (number of objects/pictures correctly named per minute)

More detailed information on these phenotypic measures, including psychometric tests used and statistical elaboration, is reported in the Supplementary Methods

### Genotype quality control (QC) and imputation

Individuals were genotyped using Illumina HumanHap 300 k, 550 k, 660 k, HumanOmniExpress, and HumanCoreExome BeadChips (see Table S2 for details). Genotype QC was carried out in PLINK v1.90b3s^46^ and QCTOOL v1.4 (see URLs), as described in Supplementary Methods and elsewhere^47^. Within each dataset, SNPs were filtered out if they showed a variant call rate < 98%; a minor allele frequency (MAF) <5%, or a Hardy–Weinberg Equilibrium (HWE) test *p*-value <10^−6^. Moreover, samples showing a genotyping rate <98%, cryptic relatedness (in datasets of unrelated subjects), identity-by-descent (IBD) not corresponding to the available pedigree information (in sibling-based datasets), and mismatches between genetic and pedigree-based sex were discarded. Furthermore, genetic ancestry outliers—detected in a multidimensional scaling (MDS) analysis of pairwise genetic distance—and samples showing significant deviations in genome-wide heterozygosity were also filtered out (see Table S3).

For imputation, autosomal variants were aligned to the 1000 Genomes phase I v3 reference panel (ALL populations, June 2014 release)^48^ and pre-phased using SHAPEIT v2 (r837)^49^. Imputation was performed using IMPUTE2 v2.3.2^50^ in 5 Mb chunks with 500 kb buffers, filtering out variants that were monomorphic in the 1000 Genomes EUR (European) samples. Chunks with < 51 genotyped variants or concordance rates < 92% were fused with neighboring chunks and re-imputed. Finally, imputed variants (genotype probabilities) were filtered out for IMPUTE2 INFO metric < 0.8, MAF < 5% and HWE test *p*-values <10, using QCTOOL v1.4. We checked again for the absence of genetic ancestry and genome-wide heterozygosity outliers after imputation, which revealed substantial concordance with pre-imputation QC. Further details on the filters used in genotype QC are reported in Table S3, while summary statistics are reported for each dataset in Table S2.

### Genetic association testing and meta-analysis

After genotype QC and imputation, autosomal genotype probabilities were tested for association with the continuous traits available within each dataset. In the datasets containing only unrelated subjects—namely AGS, Finland, France, Hungary, and The Netherlands—association with genotype dosage was tested through linear regression in PLINK v1.9, using the first 10 genetic ancestry (MDS) components as covariates. In the sibling-based datasets (Colorado and UK), a generalized linear mixed-effects model association test was carried out through FastLMM v2.07^51^, using a genetic relationship matrix (GRM) of samples as a random effect while disabling normalization to unit variance for tested SNPs.

Following separate GWAS analyses for each dataset, variant associations with each of the eight univariate traits available were combined using a fixed-effects model based on inverse-variance-weighted effect size in METASOFT v2.0.1^52^. Following the software guidelines, pooled analysis was conducted in two steps: a first run was carried out to compute genomic inflation factors, which were then used to correct meta-analysis statistics in a second run. The numbers of subjects involved in our pooled analysis were 3468 for WRead, 3399 for WSpell, 3409 for NWRead, 3093 for PA, 2591 for DigSpan, 2563 for RANlet and RANdig, and 2562 for RANpic (see Table S4 for detailed sample size by dataset). RAN measures and DigSpan were not available in the UK dataset, which was therefore not included in the pooled analyses of those traits. The numbers of variants analyzed in two or more datasets were 6,952,813 for RANlet, RANdig, RANpic, and DigSpan and 6,969,139 for WRead, WSpell, NWRead, and PA. The common genome-wide significance threshold α = 5 × 10^−8^ was corrected for multiple testing of five independent latent variables, as computed through MatSpD^53^ on the correlation matrix of the eight univariate traits analyzed (Table S1a, b). This adjustment resulted in a final Bonferroni-corrected significance level α = 1 × 10^−8^.

We also carried out a genome-wide multivariate genetic association analysis through TATES^54^, combining the univariate associations of single traits while taking into account their cross-trait correlation matrix (Table S1a). This analysis was aimed at the detection of vertical (or relational) pleiotropic genetic effects, i.e., those effects which are shared across traits due to their reciprocal relations^55^. For this analysis, the classical genome-wide significance threshold was used (α = 5 × 10^−8^).

The most significant associations detected were further investigated to assess their robustness through a permutation-based test. Moreover, we computed their effect size (regression *R*^*2*^) and tested potential epistatic effects of the variants identified. Similarly, we looked for effects of these variants on the other cognitive traits tested in our study through a horizontal pleiotropy test, aimed at detecting effects that were independent on the one observed on RANlet. Also, we looked for independent genetic effects in the genomic regions where these variants lay (18q12.2 and 8q12.3). Finally, we tested them for association with structural neuroimaging measures, which may be potentially correlated with reading and language abilities, namely subcortical volumes (see below). These analyses are reported in details in the Supplementary Methods section.

### Assessment of genes and SNPs previously associated with DD and related cognitive traits

We investigated single-variant associations for candidate SNPs and genes previously implicated in DD and related cognitive traits.

First, we assessed all the variants mapping to nine candidate genes (up to 10 kb from the 5′- or 3′-UTR): *DYX1C1*, *DCDC2*, *KIAA0319*, *C2ORF3*, *MRPL19*, *ROBO1*, *GRIN2B*, *FOXP2*, and *CNTNAP2*. For these genes, association with DD and related cognitive traits was previously reported in at least two independent studies (as reviewed in ref. ^1^). Of note, most of the candidate variants identified in these genes have been already tested in studies showing a variable degree of overlap with our cohorts (reviewed in refs. ^1,8,9^), hence they cannot be formally replicated within the scope of the current study. For this reason, we focused our replication effort on six candidate SNPs among these variants, for which a statistically significant association (*p* < 0.05 after correction for multiple testing) has been reported in the past in datasets other than ours, but was never formally replicated. These SNPs included rs6803202, rs4535189, rs331142 and rs12495133 in *ROBO1*^21,22^, rs7782412 in *FOXP2*^27^, and rs5796555 in *GRIN2B*^24^.

We next tested all the variants showing the strongest associations with DD and related cognitive traits in previous GWAS^30–35^. These included all those variants reported to be associated in previous GWAS papers, including genome-wide significant associations (*p* < 5 × 10^−8^), suggestive associations (*p* < 1 × 10^−5^), or variants reported as the most significant associations (top 10 or top 100 list, depending on the associations reported in each paper; see Results section for a complete list). Again, some of these variants were identified by studies partially overlapping with our datasets^31^, while for other SNPs tested the statistics from the original papers were not fully available or not always directly comparable, due to either different design of the study or to different traits analyzed^30–35^. Therefore, a direct comparison was possible only for few variants (see relevant Results section).

### Gene- and pathway-based enrichment tests

Gene-based association analyses for the phenotypic traits tested were performed using MAGMA v1.06^56^. First, genetic variants were assigned to protein-coding genes based on their position according to the NCBI 37.3 (hg19) build, extending gene boundaries by 10 kb from the 3′- and 5′-UTR. A total of 18,033 genes (out of 19,427 genes available) included at least one variant that passed internal QC, and were thus tested in gene-based enrichment analysis. Gene-based statistics were computed using the single-variant association statistics calculated in the GWAS of each phenotype, using default settings. To account for linkage disequilibrium (LD) among the variants tested, we used genetic data from all the datasets pooled together. Given the number of genes (18,033) and of independent latent traits (5) tested, the Bonferroni-corrected genome-wide significance threshold for this analysis was set to α = 0.05 / (18,033 × 5) = 5.5 × 10^−7^.

Using the results of the gene-based association analysis, we carried out a pathway-based enrichment test for each trait analyzed in the study, through a competitive gene-set analysis in MAGMA v1.06. We tested for enrichment 1329 canonical pathways (i.e., classical representations of biological processes compiled by domain experts) from the Molecular Signatures Database website (MSigDB v5.2, *collection C2, subcollection CP*; see URLs). To correct enrichment statistics for testing of multiple pathways, we used an adaptive permutation procedure with default settings (up to a maximum of 10,000 permutations). Hence, for gene-set analysis we corrected the significance threshold only for the number of independent latent traits tested (α = 0.05/5 = 0.01).

### Polygenic risk score analysis

To assess the genetic overlap of common variants between the dyslexia-related skills tested here and other correlated phenotypes, we carried out a polygenic risk score (PRS) analysis using PRSice v1.25^57^. This analysis tests genetic overlap between two traits by making use of GWAS summary statistics: a training GWAS is used to build the PRS, which is then tested as a linear predictor of another trait in an independent study (target GWAS). We used the eight-univariate GWAS carried out here as a target, namely WRead, WSpell, NWRead, PA, RANlet, RANdig, RANpic, and DigSpan. As training GWAS, we selected 12 different studies, involving seven subcortical volumes previously tested in a large GWAS (*N*~13,000)^58^; an educational attainment trait (expressed in years of education completed, EDUyears; *N*~293,000)^59^; and four neuropsychiatric disorders. These included ADHD (*N*~55,000)^60^; autism spectrum disorder (ASD; *N*~16,000)^61^; major depressive disorder (MDD; *N*~19,000)^62^; and schizophrenia (SCZ; *N*~150,000)^63^, and were selected in light of their comorbidity with dyslexia reported by previous literature^4,64–66^. Similarly, the choice to test subcortical volumes was driven by the increasing evidence implicating subcortical structures in reading and language abilities (as reviewed in refs. ^1,67,68^).

We performed a Summary–Summary Statistic Based Analysis using only SNPs with association *p*-values ≤ 0.05 in each training GWAS, and in linkage equilibrium (r^2^ < 0.05) with the local top hit within a 300 kb window. Only SNPs which had been tested both in the training and in the target GWAS were tested. The number of SNPs meeting these criteria ranged from 11,017 for MDD vs. DigSpan and RAN traits, to 25,409 for SCZ vs. WRead, WSpell, NWRead, and PA. To verify the robustness of our results, we repeated the analysis at increasing association significance (*P*_*T*_) thresholds in the training GWAS (with *P*_*T*_ = 0.001, 0.05, 0.1, 0.2, 0.3, 0.4, 0.5, 0.6, 0.7, 0.8, 0.9, 1.0).

To have an indication on the concordance of shared genetic effects for each pair of traits, we selected variants with association *p*-values ≤ 0.05 in each training GWAS and computed Pearson’s correlation of effect sizes (hereafter called r_β_) with each of the target GWAS analyzed. The significance threshold for these analyses was corrected for multiple testing of five independent target GWAS (i.e., the number of independent latent traits computed through MatSpD, see Table S1b), 12 different training GWAS and 12 different P_T_ thresholds tested (α = 0.05/(5 × 12 × 12) = 6.94 × 10^−5^.

## Results

For each analysis presented below, we report the empirical *p*-values, along with significance thresholds adequately corrected for multiple testing (see Subjects and methods section).

### Single-variant genome-wide associations

**Fig. 1:**
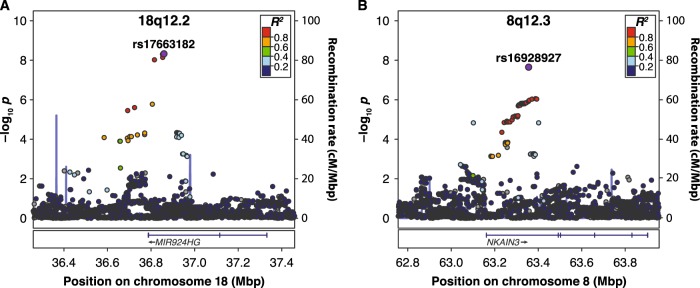
Regional association plots of lead variants. Regional association plots of **a** 18q12.2 and **b** 8q12.3 with the RANlet trait**.** The most significantly associated variants are highlighted in violet. Plots were made using LocusZoom v0.4.8^112^

**Fig. 2:**
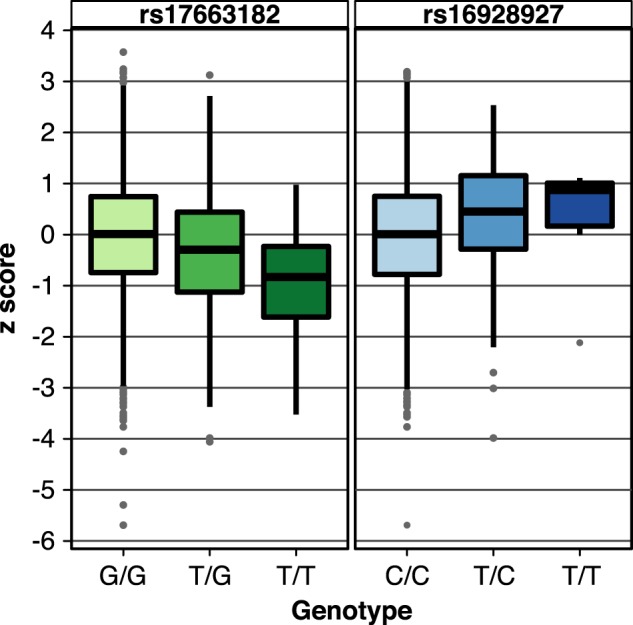
Boxplots of RANlet trait for lead variants. Boxplots of the RANlet trait as a function of genotype of the lead variants rs17663182 (left side, major allele G) and rs16928927 (right side, major allele C). Genotype counts are G/G = 2,092; T/G = 307; T/T = 16; missing = 148 for rs17663182 and C/C = 1,965; T/C = 259; T/T = 7; missing =332 for rs16928927 (Note: missing counts include Finland, where rs16928927 was not available). To generate these plots, all datasets were pooled together. RANlet Z-scores plotted here are residualized against the first 10 MDS covariates in all datasets except for Colorado, where we adjusted the phenotypic measure for pairwise genetic relatedness in GenABEL^113^ (see Supplementary Methods section)

Among the eight traits analyzed in the present GWAS, only RANlet showed genome-wide significant associations withstanding correction for multiple testing (*p* < 1 × 10^−8^), mapped to chromosome 18q12.2. The most significant association was observed for rs17663182 (G/T; MAF = 7.7%; *p*-value = 4.73 × 10^−9^, major allele (G) β (SE) = 0.35 (0.06)). All the SNPs significantly associated on 18q12 were located within the non-coding gene *MIR924HG* (*micro-RNA 924 host gene*, also known as *LINC00669*; see Fig. 1a) and were in high LD with each other (*r*^*2*^ > 0.9). An additional, independent association approaching genome-wide significance was observed with RANlet at rs16928927 (C/T; MAF = 6.5%; *p*-value = 2.25 × 10^−8^, major allele (C) β (SE) = −0.4 (0.07)) on 8q12.3. This SNP was located within the first intron of *NKAIN3* (*Na+/K+ transporting ATPase interacting 3*; see Fig. 1b). Further details on these associations are reported in Fig. 2 and Table 3.

**Table 3:** Most significant single-variant associations (*p* < 1 × 10^−7^) detected in the univariate GWAS analyses

SNP	A1	A2	A1 frequency^a^	*p*-value	β^b^	β SE	I^2 c^	Location (chr:bp)	LD relative to local top hit (*r*^*2*^)	Gene symbol	Position relative to gene	Distance from gene (bp)	Trait
rs17663182	G	T	0.92	4.73 × 10^−9^	0.353	0.060	0	18:36859202	–	*LINC00669*	intronic	–	RANlet
rs17605546	G	A	0.92	4.92 x 10^−9^	0.352	0.060	0	18:36852398	0.98	*LINC00669*	intronic	–	RANlet
rs74500110	C	T	0.92	7.14 x 10^−9^	0.343	0.059	0	18:36853535	0.94	*LINC00669*	intronic	–	RANlet
rs34822091	G	A	0.92	9.44 × 10^−9^	0.347	0.060	0	18:36815582	0.94	*LINC00669*	intronic	–	RANlet
rs16928927	C	T	0.94	2.25 × 10^−8^	−0.403	0.072	0	8:63356625	–	*NKAIN3*	intronic	–	RANlet
rs1541518	G	T	0.71	6.42 × 10^−8^	−0.177	0.033	0	7:31148279	–	*ADCYAP1R1*	downstream/3´-UTR	1956	NWRead

^a^Average allele frequency computed over all the datasets analyzed

^b^β values are relative to A1

^c^I-squared test for heterogeneity of genetic effect across datasets (the closer to “0”, the more homogenous is the genetic effect)

**Fig. 3:**
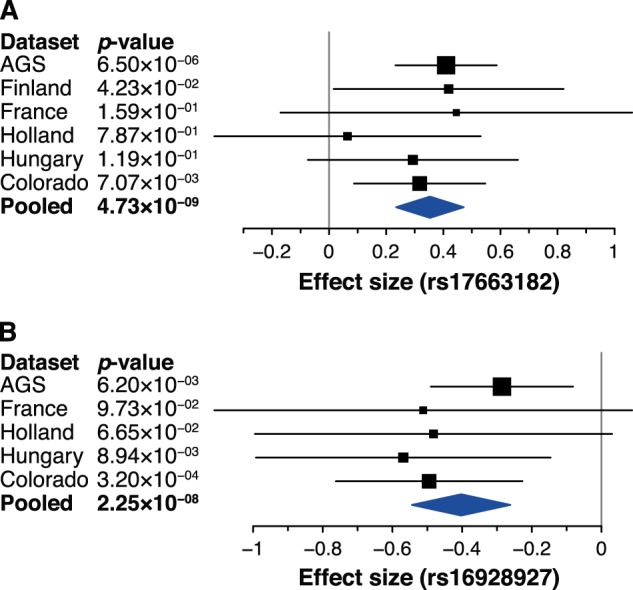
Forest plot of associations of lead variants with RANlet. Forest plots of association signals with RANlet for **a** rs17663182 (18q12.2) and **b** rs16928927 (8q12.3). Effect sizes (β) refer to major alleles **a** G and **b** C, respectively

**Fig. 4:**
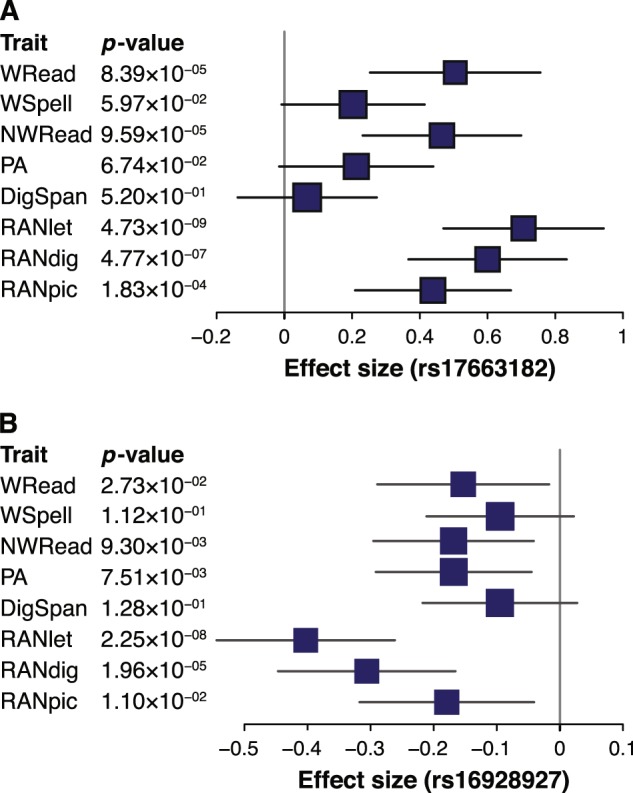
Forest plots of multi-trait associations for lead variants. Forest plots of associations of **a** rs17663182 (18q12.2) and **b** rs16928927 (8q12.3) with the different traits analyzed in the study. Effect sizes (β) refer to major alleles **a** G and **b** C, respectively

Although neither of the two top SNPs was genotyped, imputation quality was high in all datasets (IMPUTE2 INFO metric 0.89–0.94 for rs17663182 and ~0.99 for rs16928927, respectively). These variants showed consistent allelic trends (Fig. 3a, b), but explained a variable proportion of RANlet variance in the different datasets ([0.03–1.8]% for rs17663182 and [0.067–2.96]% for rs16928927, respectively; Table S5a, b). Both our lead SNPs showed evidence of an association with many of the traits analyzed, especially with RAN traits (see Fig. 4a, b). Indeed, a genome-wide multivariate association analysis with the eight cognitive skills detected a significant association at rs17663182 (*p* = 3.07 × 10^−8^), and a suggestive association at rs16928927 (*p* = 1.46 × 10^−7^). Similarly, a multivariate association test focused on the three RAN traits revealed a genome-wide significant association of rs17663182 (*p* = 1.15 × 10^−8^), while rs16928927 association only approached significance (*p* = 5.45 × 10^−8^). However, neither of these two SNPs showed significant effects independent from RANlet on any other trait (Table S5c, d). Similarly, we observed no significant independent genetic influence on RANlet at 18q12.2 and 8q12.3, in a 100 kb window surrounding rs17663182 and rs16928927 (Table S5e, f), as well as no significant epistatic effect of these two variants on RANlet (Table S5g). These SNPs did not show any statistically significant association with volumes of seven different subcortical structures (Table S5h, i).

Detailed results of the GWAS analyses for each univariate trait are reported in Supplementary Figure S1a–p and Table S6a–h, while the most significant multivariate associations detected genome-wide are reported in Table S6i.

### Genes and SNPs previously associated with DD and related cognitive traits

In total, 12,785 variants were annotated to nine candidate genes previously implicated in dyslexia by at least two independent studies, namely *DYX1C1*, *DCDC2*, *KIAA0319*, *C2ORF3*, *MRPL19*, *ROBO1*, *GRIN2B*, *FOXP2*, and *CNTNAP2*. We reported associations for all these variants in Table S7a–h. Among these variants, a detailed assessment of six candidate SNPs previously associated with DD or related cognitive measures in independent studies did not reveal any strong evidence of replication in our cohorts (see Table S7i), although we found marginal evidence of association of the *ROBO1* variant rs12495133 with WSpell (C/A; MAF = 40%; *p*-value = 0.045, major allele (C) β (SE) = −0.06 (0.03)), with an allelic trend concordant with the original report^22^.

Similarly, among variants associated with DD and related cognitive measures in previous GWAS efforts (see Table S8a–i), we identified a few nominally significant associations (*p* < 0.05) that were comparable with those reported by previous independent studies (Table S8j). The most significant associations were observed at rs10485609, an intronic SNP located within the *CSE1L* gene (20q13.13), with both word (A/G; MAF = 12%; *p*-value = 2.6 × 10^−3^, major allele (A) β (SE) = −0.12 (0.04)) and nonword reading (*p*-value = 6.5 × 10^−3^, major allele (A) β (SE) = −0.1 (0.04)). These associations showed the same direction of effect as in the original report^34^.

### Gene- and pathway-based associations

Gene-level analyses of single-variant association signals in MAGMA revealed no significant enrichment of genes after correcting for testing of 18,033 protein-coding genes and of five independent latent traits (α = 5.5 × 10^−7^; see Table S9a–h). The most significant association was observed for the gene *ADCYAP1R1* (*adenylate cyclase activating polypeptide 1 receptor type I*; 7p14.3) with NWRead (Z-score = 4.6; *p* = 2 × 10^−6^). Similarly, also in the gene-set analysis of 1329 canonical pathways from the MSigDB website, no pathway was significantly enriched (α = 0.01 for permutation-based enrichments, already corrected for testing of multiple pathways; see Table S10a–h). However, we found a nominally significant enrichment of associations with WSpell for genes in the BioCarta RAS pathway (Bonferroni-corrected *p* = 0.045; β (SE) = 0.64 (0.16); see Table S10i for a complete list of genes leading the pathway-based association).

### Genetic overlap with neuroimaging, neurodevelopmental, and neuropsychiatric phenotypes

**Fig. 5:**
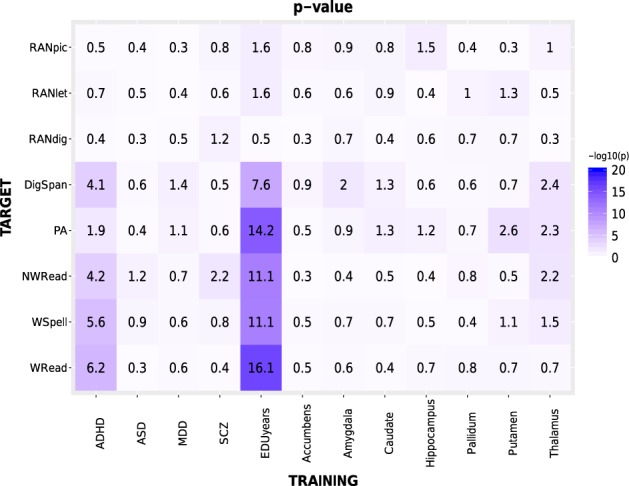
Polygenic Risk Score analysis. Results of the polygenic risk score (PRS) analysis on the eight traits analyzed in this work (target traits), which were compared with different neuropsychiatric, educational, and neuroimaging phenotypes (training traits). In the heatmap, –log(*p*) of the *R*^*2*^ computed by PRSice^57^ at an association *p*-value threshold (P_T_) of 0.05 is reported. Complete summary statistics are reported in Tables S11a, b, c

PRS analysis revealed the presence of a significant proportion of shared genetic variance between the different DD-related traits analyzed in our GWAS and some of the neuroimaging, educational, and neuropsychiatric phenotypes investigated in previous large GWAS studies (see Fig. 5; Table S11a–c). In particular, we observed significant genetic overlaps withstanding Bonferroni correction (*p* < 6.94 × 10^−5^) with ADHD risk, and with educational attainment (EDUyears). The ADHD PRS was negatively associated with WRead, WSpell, and NWRead (at PT = 0.05: *Nagelkerke’s R*^*2*^ ranging from 0.004 for NWRead to 0.007 for WRead; *p* ~ [10^−5^–10^−7^]), while EDUyears polygenic score was positively associated with WRead, WSpell, NWRead, DigSpan, and PA (at PT = 0.05: *R*^*2*^ ranging from 0.011 for DigSpan to 0.019 for WRead and PA; *p* ~ [10^−8^–10^−17^]). These results were substantially confirmed at different *P*_*T*_ thresholds (see Figure S11a–h).

## Discussion

In the present study, we investigated genetic effects on eight different neurocognitive skills, including behavioral features and predictors of dyslexia. We conducted a GWAS of up to 3468 subjects from nine different countries, speaking six different languages. Hence, our study represents the most detailed GWAS in the field in terms of phenotypes investigated, countries and languages involved, and one of the largest reported so far.

We identified a genome-wide significant effect on rapid automatized naming of letters, which showed a (relational) pleiotropic influence on the whole RAN domain and, to a lesser extent, on reading abilities. Rapid naming reflects the automaticity of visual–verbal access necessary for efficient word decoding, and accounts for a significant proportion of variance in word reading ability, especially reading fluency, which is independent of the well-established language and phonological processes implicated in reading, like phoneme awareness^69^. This association between RAN and reading (fluency) has been reported across different orthographies^45^ and over the life span^70^. Furthermore, RAN turned out to be a significant predictor of poor reading skills across orthographies^38^ and is also used in kindergarten to identify children at risk of dyslexia^71^. Good predictivity of reading performance has been reported for both alphanumeric and non-alphanumeric RAN measures, although correlations are usually stronger between alphanumeric RAN and reading fluency, compared with non-alphanumeric RAN (as reviewed by Kirby et al.^72^). The correlations between alphanumeric (letters and numbers) RAN and reading skills are also significant through adulthood^73^. Importantly, RAN predicts later reading performance independently from reading experience or early differences in reading ability^74–76^, and from phoneme awareness. While RAN has been shown to be an important predictor for reading problems, it should be noted that, in line with multiple-deficit models of dyslexia^77^, RAN represents one of several predictors of DD risk and reading abilities (reviewed in ref. ^78^). More recently, RAN has been also associated with other learning disorders, like dyscalculia, and it has been hypothesized that RAN deficits in dyslexic children may be independent from those detected in dyscalculic children^79,80^.

The most significant association signal with RANlet was observed for rs17663182, a variant located within *MIR924HG* (18q12.2; *micro-RNA 924 host gene*, or *LINC00669*). Additional significant associations were detected in the same region for other variants, all in high LD with the lead SNP, which suggests that they identified the same genetic effect on RANlet. This observation was supported by the absence of strong independent genetic effects on RANlet within a 100 kb window surrounding the strongest signal at rs17663182. An extensive lookup of these 18q12.2 variants in common online gene expression databases—including the Genotype-Tissue Expression portal (GTEx)^81^, the Brain eQTL Almanac (Braineac)^82^, the Blood eQTL browser^83^, and the seeQTL database^84^ —revealed weak evidence of expression quantitative trait loci (eQTL) involving rs17663182 and neighboring associated SNPs. Braineac reports nominally significant eQTL effects (*p*-value < 0.05) for these SNPs on *MIR924HG* expression in the occipital cortex, thalamus, and substantia nigra. In addition, HaploReg v4.1 indicated the presence of histone marks usually associated with transcriptional activity in the same region, such as H3K4me1, H3K27ac, and H3K9ac^85^. To the best of our knowledge, no regulatory role is known for *MIR924HG*, and *MIR924* has not been functionally characterized so far. Nonetheless, the significant associations on 18q12.2 represent an interesting genetic effect for three main reasons:

First and foremost, evidence of genetic linkage to dyslexia-related cognitive traits has been reported for this region in previous studies, although not always reaching statistical significance^86–89^. In a genome-wide linkage analysis of a German cohort partly overlapping with our AGS dataset, a linkage peak to a principal component of RAN scores was observed in a region encompassing the microsatellite marker *D18S1102*, located ~2.1 Mb downstream of rs17663182^89^. Similarly, a linkage signal was later reported for the same marker with a composite RAN score, in a Dutch sib-pair sample. However, this association was weaker after including parents of the sib-pairs in the analysis^86^. Early evidence for linkage in 18q12 has been reported with word reading and orthographic coding, in samples partially overlapping with our Colorado and UK datasets^87,88^. In line with these findings, rs17663182 showed associations with traits other than RANlet in our analysis, including RANdig, RANpic, WRead, and NWRead (further discussed below). It would be tempting to connect the linkage signals mentioned above with the SNP associations at rs17663182, but it is important to point out that this association likely represents only a small fraction of these linkage signals or even a distinct genetic effect, because linkage and association analyses tend to detect different effects^90^.

Second, a search for binding sites through the online database TargetScanHuman v7.1^91^ allowed us to identify a series of interesting candidate target genes which *MIR924* could regulate. These include candidate dyslexia susceptibility genes like *MRPL19* and *KIAA0319L*, although these did not show the highest predicted binding scores to *MIR924* (cumulative weighted context++ scores −0.08 and −0.07; ranked 1615 and 1626 over 3472 potential targets).

Third, *MIR924HG* is expressed in a number of cancer cell lines, but consistently in samples representing iPS differentiation into neurons, according to the FANTOM5 miRNA promoter analysis^92^. This is interesting in the context that at least three dyslexia candidate genes (namely *DCDC2, DYX1C1*, and *KIAA0319*) have been implicated in regulating neuronal migration and cilia functions in model systems^9^.

In the analysis of RANlet, we observed an additional association approaching genome-wide significance at rs16928927 (8q12.3). This intronic variant is located within *NKAIN3* (*Na+/K+ transporting ATPase interacting 3*), a gene which is widely and specifically expressed in the brain, especially in the fetal temporal lobe, in newborn and in adult hippocampal regions^93^.

Of note, both our lead SNPs showed associations with different cognitive measures analyzed in this study, especially with RAN traits. This multitrait association trend is particularly noticeable for rs17663182, which showed convincing evidence of influence within and even beyond the RAN domain, extending to reading abilities, as suggested by a genome-wide significant multivariate association with all the cognitive traits analyzed. However, a horizontal pleiotropy test on both variants did not reveal any significant effect specific to cognitive traits other than RANlet. This suggests that these variants likely exert their genetic influence on the common phenotypic variance underlying these traits, with different magnitude of effect on each measure, rather than on trait-specific phenotypic variance.

Despite the biological appeal of the top association signals mentioned above, an imaging genetic assessment of these SNPs did not reveal any significant effect on variation in seven different subcortical volumes^58^. Considering the sample size of this neuroimaging genetic analysis (*N*~13,000), we deem it unlikely that this lack of support is caused by a lack of power. However, this negative result does not rule out genetic effects of the variants detected here on other brain structures involved in reading networks, such as the inferior frontal gyrus and the temporal and parietal gyri. These potential associations should be tested in the future, as was previously done for other variants associated with reading-related traits^94,95^.

An assessment of candidate genes and SNPs implicated in dyslexia and related traits by other studies provided weak or (in most cases) no evidence of replication. Several possible factors may account for these apparently contrasting results. First, the heterogeneity of recruitment of the samples analyzed may lead to discrepant results across different studies: some genetic variants may have stronger effects in the lower tail of the reading and language skills distributions (i.e., in selected DD samples) and negligible effects in a broader range of variation (i.e., in general population samples). Second, the heterogeneity of assessment of the phenotypes may result in traits that ostensibly tap into the same cognitive domain but actually represent slightly different abilities. This applies not only to continuous DD-related measures, but also to the classification of dyslexia cases and controls, for which a consensus is far from being reached in the scientific community^3,96,97^. Third, different genetic backgrounds of the populations analyzed may be a factor when comparing or meta-analyzing different association studies. The haplotype structure in a specific region may differ between populations, and so may change the LD between the tag SNP (where the association is detected) and the genuine causal SNP (which determines the association). In the presence of substantial population stratification, this could even result in contrasting directions of effect for the same SNP in different studies^37,98,99^. Fourth, the inconsistent results from association studies may be due to different age ranges of the samples analyzed, e.g., when comparing an adult population with a datasets made up of children^37^. An alternative explanation may be that the original findings were type I errors, since false-positive results may easily occur in analyses of relatively small samples^100^. While this is a less likely explanation for those associated SNPs which have been functionally investigated, it may reasonably account for spurious associations, which are more likely to be affected by publication biases (i.e., significant results tend to be favored for publication) and reporting biases (i.e., investigators tend to report only positive findings).

Another interesting finding of our study is the significant genetic overlap that some of the traits analyzed showed with educational attainment (EDUyears) and ADHD. Educational attainment was already reported to share a significant proportion of genetic variance with word reading ability^101,102^. In a PRS analysis comparing educational attainment with reading efficiency and comprehension, the same EDUYears score used here^59^ accounted for 2.1% (at the age of 7) to 5.1% (at the age of 14) of the variance in such reading measures in a UK sample (*N* = 5825), and this association remained significant even after correcting for general cognitive ability and socioeconomic status^101^. More recently, Luciano et al.^102^ used the results of a previous GWAS on reading and language-related traits^32^ to test genetic correlations with several health, socioeconomic, and brain structure measures collected in adults from the UK (maximal *N* = 111,749; age range 40–69 years). Polygenic scores increasing these traits—namely word reading, nonword repetition, and a reading–spelling score— were all positively associated with a binary index of educational attainment (college or university degree)^102^. In our paper, we replicate these findings by reporting that variants nominally associated with EDUyears explain almost 2% of the total variance in WRead (used here as a target trait), and extend the evidence of genetic overlap to other behavioral features—WSpell and NWRead—and to cognitive predictors of dyslexia risk like PA and DigSpan.

Our PRS analysis also revealed a shared genetic basis for ADHD risk and the core dyslexia features WRead, WSpell, and NWRead. This long-standing hypothesis was originally supported by behavioral genetics studies of twins^103–105^, and has been later corroborated by molecular genetic studies. The existence of overlapping risk loci between DD and ADHD suggests that these regions could be the potential sites of liability underlying ADHD–DD comorbidity^1^. Candidate DD susceptibility genes like *DYX1C1*, *DCDC2*, and *KIAA0319* have been associated with inattention and hyperactivity/impulsivity^106–108^, and candidate ADHD genes like *DRD4* have been investigated for linkage and association with DD, with inconsistent results^109,110^. More recently, Mascheretti et al.^111^ found significant main and interactive associations upon hyperactivity/impulsivity involving *DCDC2* and *KIAA0319*, while Sánchez-Móran et al.^25^ reported stronger associations of candidate *KIAA0319* and *FOXP2* variants with ADHD–DD comorbid cases, compared with simple dyslexic subjects. In line with this evidence, our findings provide further support to a partly shared genetic etiology of DD and ADHD at the genome-wide level.

Among the limitations of our study are a certain variability in the inclusion criteria and phenotypic assessment of some cohorts^18^, the absence of a follow-up cohort to replicate the genome-wide significant associations detected, and the modest power to detect small effect sizes (see Supplementary Methods). These are counterbalanced by strengths of our study, which include the variety of continuous neurocognitive traits analyzed, covering all the most relevant dyslexia-related behavioral phenotypes, and the homogeneity of QC procedures among datasets, which are fundamental to improve statistical power. Indeed, most of our samples were collected in the context of a large international consortium for studying the neurobiological/genetic basis of dyslexia (Neurodys), whose main purpose is to homogenize traits and datasets to allow for comparable analyses across different countries^38,42^. Overall, this study represents an early step of one of the largest international collaborations aimed at clarifying the genetic basis of reading abilities and disabilities, which will hopefully contribute to shed a light on the neurobiology of dyslexia.

## URLs

Human Integrated Protein Expression Database: http://www.genecards.org/. FANTOM5 Zenbu database: http://fantom.gsc.riken.jp/zenbu/. PLINK: https://www.cog-genomics.org/plink2. QCTOOL: http://www.well.ox.ac.uk/~gav/qctool/. MatSpD: http://gump.qimr.edu.au/general/daleN/matSpD/. MAGMA: http://ctg.cncr.nl/software/magma. MSigDB: http://software.broadinstitute.org/gsea/msigdb; PRSice: http://prsice.info/. Genotype-Tissue Expression portal (GTEx): http://www.gtexportal.org/home/. Brain eQTL Almanac (Braineac): http://www.braineac.org/. Blood eQTL: http://genenetwork.nl/bloodeqtlbrowser/. seeQTL: http://www.bios.unc.edu/research/genomic_software/seeQTL. HaploReg: http://archive.broadinstitute.org/mammals/haploreg/haploreg.php. TargetScan: http://www.targetscan.org/. LocusZoom: http://www.locuszoom.org/

## Supplementary information

Suppementary Methods

Supplementary Results: Manhattan and QQ plots

Supplementary Results: Characterization of top association signals

Supplementary Results: Most significant single variant associations (GWAS)

Supplementary Results: Associations in candidate dyslexia susceptibility genes

Supplementary Results: Associations of candidate SNPs from previous GWAS

Supplementary Results: most significant gene-based associations

Supplementary Results: most significant pathway-based associations

Supplementary Results: PRS analysis
