# Supplementary material for: Genome-wide association scan identifies new variants associated with a cognitive predictor of dyslexia
Source: Transl Psychiatry. 2019 Feb 11;9:77. doi: 10.1038/s41398-019-0402-0 (PMC6370792; doi:10.1038/s41398-019-0402-0)
Supplement: Supplementary file 1 — Suppementary Methods [file 41398_2019_402_MOESM1_ESM.pdf]

Supplementary Methods

Phenotypic Measures

Word Reading (WRead)

In Austria, Germany, Switzerland, Finland, France, Hungary, and the Netherlands, reading accuracy and speed for words were assessed by presenting language-specific material under a speeded instruction ("Read as quickly as possible without making mistakes"). The number of words read correctly per minute was then converted into z-scores based on grade-appropriate norms, separately in each country (see [1,2] for details). In the UK dataset, an untimed word reading task was administered, which was grade-normed and standardized as above. In the Colorado dataset, a composite (average) measure of timed and untimed word reading was used, which was age-adjusted and standardized against the normative mean of a matching control population (see [3] for details).

Word Spelling (WSpell)

Language-specific standardized spelling tests were administered in each country. In all the European countries, spelling tests required to spell single words dictated in sentence frames[1]. Grade-specific z-scores for the percentage of words spelled correctly were calculated based on language-specific norms in all datasets except Colorado, where the spelling test consisted of choosing the correct spelling for a series of real words orally presented and the measure was age-adjusted and standardized against a matching control population[3].

Nonword Reading (NWRead)

In all non-English-speaking countries nonword reading was tested under a speeded instruction, using language-specific material. The relevant measure -namely the total number of items correctly read per minute- was grade-normed within each country, as done with the word reading task. In UK and Colorado an untimed nonword reading task was administered. The resulting raw scores were then grade-normed and age-adjusted in the two countries, respectively, as explained above[1–3].

Phoneme Awareness (PA)

Phoneme awareness is defined as the ability to manipulate the smallest pronounced unit in a word (i.e., the phoneme). In all countries except for UK and Colorado, this was assessed through a phoneme deletion test. A typical task of this kind consists of pronouncing a sound sequence after deleting a specified sound (e.g. say *"/gulst/without/l/"*). Language-specific tasks were constructed with comparable difficulty levels in all countries, to account for different orthography consistency[1]. In the UK dataset, PA was assessed through phoneme deletion and substitution in single words and spoonerism tasks (i.e. swapping the first sounds of two words, e.g. from *spoon, dog* to *doon, spog*). In all the countries mentioned above, raw scores were grade-normed and standardized as before. In Colorado, PA was assessed through i) a classical phoneme deletion task and ii) a phoneme segmentation and transposition task, i.e. taking the first phoneme of a word, putting it at the end and adding the sound "/ay/" (e.g. *rope* becomes *ope-ray*). Then an average score of these tests was computed, which was age-adjusted and standardized against a matching control population[3].

Digit Span (DigSpan)

Digit Span was tested in all countries except the UK through a classical WISC (Wechsler Intelligence Scale for Children) digit span task[4,5]. Such a task consisted of reciting a sequence of digits visually presented by recalling them in the same (forward) and reverse (backward) order. Raw scores (sum of forward and backward test scores) were then converted into scaled scores (mean = 10, SD = 3) and finally into z-standardized scores based on national norms within each country[1,2]. However, in this case no grade-normalization was carried out[1].

Rapid automatized naming (RAN)

RAN tests were administered in all countries except the UK. Children were asked to name as quickly and as accurately as possible a matrix of digits (RANdig), letters (RANlet), and objects/pictures (RANpic) that was visually presented. The resulting raw score (i.e., the number of items correctly named per minute) was then grade-normed and standardized as above in all the European countries[1,2], while it was age-adjusted and then standardized against a control population in Colorado[3].

Phenotypic outlier detection

To check for the presence of phenotypic outliers in our datasets, we defined them within each dataset as subjects showing phenotypic z-scores at least 4 standard deviations (SDs) below or above the mean for the majority of the traits available within each dataset (i.e., in at least 3 out of 4 traits in the UK dataset and in at least 5 out of 8 traits in all the other datasets). None of the subjects tested met this condition in any dataset.

Genotype quality control (QC) and imputation

Quality control of genotyped data was conducted in PLINK 1.90b3s[6]. Some of the QC filters applied differed between the sibling-based datasets analysed (i.e., UK and Colorado) and the cohorts made up of unrelated subjects (AGS, Finland, France, Hungary, and the Netherlands), as reported in detail in Table S2. Prior to imputation, genotypes were aligned to the 1000 genomes phase I v3 reference panel (June 2014 release)[7] using SHAPEIT v2 (r837)[8] and PLINK v1.90b3s. Subsequently, pre-phasing (haplotype estimation) was conducted for each chromosome separately using SHAPEIT. Imputation was performed using IMPUTE2 v2.3.2[9] in 5 Mbp chunks with 500 kbp buffers, filtering out variants that were monomorphic in the EUR samples. Chunks with < 51 genotyped variants or concordance rates < 92 % were fused with neighbouring chunks and re-imputed. After imputation, variants (genotype probabilities) were filtered for MAF $\geq$ 5 %, IMPUTE2 INFO metric $\geq$ 0.8, and HWE test p-values $\geq 10^{-6}$ using QCTOOL v1.4 (see URLs).

Trait	WRead	WSpell	NWRead	PA	DigSpan	RANdig	RANlet	RANpic
WRead	1	0.665	0.84	0.543	0.298	0.541	0.595	0.486
WSpell	0.665	1	0.584	0.536	0.322	0.373	0.389	0.366
NWRead	0.84	0.584	1	0.531	0.285	0.557	0.597	0.463
PA	0.543	0.536	0.531	1	0.35	0.327	0.361	0.326
DigSpan	0.298	0.322	0.285	0.35	1	0.173	0.184	0.209
RANdig	0.541	0.373	0.557	0.327	0.173	1	0.722	0.591
RANlet	0.595	0.389	0.597	0.361	0.184	0.722	1	0.578
RANpic	0.486	0.366	0.463	0.326	0.209	0.591	0.578	1

Table S1a. Cross-trait correlations (Pearson's *r* coefficient) of the eight cognitive skills analysed in the present study. These coefficients are based on the seven datasets made up of unrelated subjects (AGS, Finland, France, Hungary, and the Netherlands; see Table 1 in the main text and Table S2 below), while for the sibling-based datasets (Colorado and UK) cross-trait correlations can be found in Gialluisi et al.[3] (see Table S4a, c).

VAR	**1**	**2**	**3**	**4**	**5**	6	7	8
WRead	**-0.8761**	**0.0963**	**-0.2424**	**0.2118**	**-0.0457**	0.1845	-0.0066	0.2889
WSpell	**-0.7293**	**0.347**	**-0.2538**	**-0.0069**	**-0.3813**	-0.3643	0.0401	-0.0599
NWRead	**-0.858**	**0.0548**	**-0.2271**	**0.248**	**0.0769**	0.2721	-0.0781	-0.2484
RANlet	**-0.7787**	**-0.4066**	**0.0992**	**0.0869**	**0.2077**	-0.1393	0.3847	-0.0163
RANdig	**-0.751**	**-0.4513**	**0.1535**	**0.0407**	**0.1505**	-0.2574	-0.3427	0.0249
RANpic	**-0.6917**	**-0.3603**	**0.2839**	**-0.3431**	**-0.3703**	0.2367	0.0108	-0.0149
PA	**-0.6709**	**0.4188**	**-0.124**	**-0.4957**	**0.3363**	0.0115	-0.01	0.0104
DigSpan	**-0.4207**	**0.5691**	**0.6812**	**0.1864**	**0.0187**	0.0024	0.0015	0.0025

Table S1b. Latent variables computed through a Principal Component Analysis (PCA) of the cross-trait correlation matrix (see Table 1a above), performed in MatSpd[10]. Here, PC coefficients of each trait on the latent variables computed for the unrotated matrix are reported. We report in bold those latent variables (VAR1 to VAR5) which were considered in the correction for multiple phenotypic traits tested, as computed by MatSpd (see main text). Overall, these variables explained 90% of the total shared variance in the cognitive traits analysed.

Dataset	N	N After QC	Genotyping platform	SNPs	SNPs After QC	SNPs after imputation
AGS	1,502	1,454	Illumina HumanHap 300k	317k [a]	292,056[a]	5,747,699
			Human CoreExome	543k	240,130	
Finland	336	324	Human CoreExome	543k	243,282	6,197,696
France	165	163	Illumina HumanHap 660k	660k	471,468	6,177,173
Hungary	243	241	Human CoreExome	543k	236,643	5,948,732
Netherlands	311	284	Human CoreExome	543k	236,871	5,978,155
Colorado	585	550	Illumina Human OmniExpress	683k [b]	545,749	6,220,298
UK	983	923	Illumina HumanHap 550k	310k [b, c]	277,931	6,040,246
			Human OmniExpress			

Table S2. Main genotype QC statistics and information for each dataset involved in the study.

[a] A subset of the German sample (N=195) was genotyped on the Illumina 317k chip and shared a low number of SNPs with the Human CoreExome array. These samples were thus QCed separately and merged with the rest of the AGS samples only after imputation.

[b] SNPs in these datasets were preliminarily filtered through Illumina GenomeStudio software before producing hard-call genotype data, as described by Gialluisi et al.[3].

[c] Since this dataset was genotyped on two different platforms, only variants which were shared between the two arrays were used in the following genotype QC and imputation.

Filter	Sibling-based datasets	All other datasets
Individual genotyping rate	> 98 %	
Genetic relatedness (PI-HAT)	among unrelated subjects: PI-HAT < 0.2; among related subjects: PI-HAT in the range [0.2 ; 0.65[and concordant with pedigree based information [a]	< 0.0625
Duplicates (PI-HAT)	≤ 0.65	
Sex mismatches with pedigree-based info	based on X chromosome genetic data (PLINK v1.9 default settings)	
Genetic ancestry: distance in first two MDS components from mean [b, c]	< 4 SD	< 5 SD
Genome-wide heterozygosity: deviation of autosomal heterozygosity from mean [c]	< 3 SD	< 4 SD
Minor allele frequency [c]	≥ 5 %	
Variant call rate [c]	≥ 98 %	
HWE test *p*-value [c]	$\geq 10^{-6}$	
Variants on non-autosomal chromosomes	removed	
Ambivalent SNPs (A/T and G/C)	removed	
Variants not present in the 1000 genomes phase I v3 EUR reference panel	removed	
IMPUTE2 info metric	≥ 0.8	

Table S3. Details on genotype QC and imputation of the datasets involved in the study. Sibling-based datasets: Colorado and UK; all other datasets: Austria-Germany-Switzerland (AGS), Finland, France, Hungary and the Netherlands.

[a] In sibling-based datasets, samples showing PI_HAT values discordant with pedigree-based information were filtered out (i.e. unrelated subjects showing PI-HAT ~0.25-0.5; related subjects showing PI-HAT <0.2; and subjects showing PI-HAT ~1).

[b] MDS components of the genetic distance matrix were calculated through PLINK 1.9. First, we used the QCed and imputed genetic data to build an IBS similarity matrix for all the subjects within each dataset (through the --genome command); then we extracted the first 10 MDS components from the IBS matrix (through the --cluster --mds-plot commands).

[c] These filters were applied both before and after imputation.

Dataset	Passing QC	Wread	Wspell	NWRead	PA	DigSpan	RANdig	RANlet	RANpic
AGS	1454	1127	1116	1126	1126	1127	1126	1126	1126
Finland	324	324	320	300	324	324	323	323	323
France	163	143	120	120	119	142	120	120	120
Hungary	241	236	236	236	234	236	236	236	236
Netherlands	284	232	228	230	225	230	225	225	224
Colorado	550	533	527	529	527	532	533	533	533
UK	923	873	852	868	538	0	0	0	0
Meta	3939	3468	3399	3409	3093	2591	2563	2563	2562

Table S4. Sample size (after QC) of the datasets involved in the study, including number of subjects analysed for each continuous trait.

Power and sample size estimation analysis

We performed a sample-size estimation analysis using the Genetic Power Calculator[11]. Specifically, we computed the sample size (N) required to detect genome-wide significant associations with continuous traits, assuming a 50:50 dominance-to-additive QTL ratio, and a QTL with MAF = 5% and in perfect LD with the causative SNP, which could explain 1% of the total phenotypic variance (i.e. $R^2 = 0.01$). Since some of our cohorts were made up of unrelated subjects, while others were sibling-based, we performed the analyses in both alternative settings. Assuming a dataset of unrelated subjects, a sample size N=4,280 was required to have a 80% power to detect genome-wide significant associations ($p < 5x10^{-8}$), and N=4,646 to detect associations surviving our correction for multiple traits tested ($p < 1x10^{-8}$, see Table S1b above and main text). On the other hand, under the assumption of a sibling-based cohort (with sibling correlation of 0.5), the sample sizes required were N=2,136 and N=2,319, respectively. Assuming a QTL with effect size $R^2 = 0.005$, the samples sizes required to reach a power of 80% were N=8,582 (for association $p < 5x10^{-8}$) and N=9,316 (for $p < 1x10^{-8}$) in the unrelated subjects settings, and N=4,283 and N=4,649 in the sib-pairs settings. Due to the mixed nature of our cohorts –with some datasets made up of unrelated subjects and others made up of sibling pairs or, rarely, trios – it is likely that the sample size required to have a 80% power in our GWAS meta-analysis is in the middle between the values reported above for the different settings.

Further analyses of top association signals

The analyses explained in this section were only conducted on datasets with RANlet measures available (see Table S4) and required the preliminary adjustment of phenotypic traits for genetic population structure in each dataset. This was carried out differently in the datasets including only unrelated subjects (AGS, Finland, France, Hungary, and The Netherlands) and in the sibling-based dataset (Colorado). In the former group, we regressed the phenotypic traits against the first ten MDS components (previously used as covariates in the GWAS). In the latter case, we adjusted the traits for a GRM through the *polygenic()* function of the GenABEL package[12].

Permutation-based correlation test and effect size estimation

To assess the robustness of the most significant associations detected (with RANlet), we carried out a permutation-based test on the top-associated SNPs at 18q12.2 (rs17663182) and 8q12.3 (rs16928927) in R v3.2.3[13]. Briefly, we first computed allelic dosages from genotype probabilities for the SNPs of interest within each dataset, and adjusted the RANlet score for genetic population structure in each dataset (as explained above). Subsequently, we computed Pearson correlation through the *cor()* function of the WGCNA v1.51 package[14]. After the calculation of the Pearson correlation coefficient r, we permuted both phenotypic residuals and dosages 10,000 times, computing similar correlation coefficients for each of the resulting 10,000 × 10,000 = 100 million random combinations. Finally, we derived an empirical p-value from the distribution of these 100 million random correlations (defined as the frequency of random correlations which were at least as high as our original correlation coefficient r).

To estimate the fraction of RANlet phenotypic variance explained by rs17663182 (18q12.2) and rs16928927 (8q12.3) within each dataset, we used R to compute linear regression R^2 of the phenotypic trait adjusted for genetic population structure vs dosage values of the top-associated variants.

Further tests of pleiotropy

In addition to the genome-wide multivariate association test across all the DD-related traits analyzed, for the two most significant univariate (and multivariate) association signals we carried out a specific multivariate association test on RAN traits only, through TATES[15]. This software combines the p-values obtained in univariate genetic association analysis on multiple (correlated) phenotypes, to produce one multivariate association p-value per SNP, while correcting for their correlations. For this analysis, we used as input the association p-values of the top hits with RANlet, RANdig and RANpic, as well as their correlation matrix (i.e. last three columns and last three rows of Table S1 above).

Moreover, we tested the top association signals for horizontal (independent) pleiotropic effects on traits other than RANlet, namely WRead, WSpell, NWRead, PA, DigSpan, RANdig and RANpic. To this end, we first regressed these traits, which had previously been adjusted for genetic population structure, against the RANlet score in R, separately for each dataset. Then we tested the residuals of these traits for association with rs17663182 and rs16928927 dosages in PLINK. Finally, we combined the results of the association tests in different datasets through an inverse-variance fixed-effect pooled analysis in METAL v25-03-2011[16], which allowed us to directly detect concordance of allelic trends across datasets for all the SNPs tested.

Test for independent genetic effects in 18q12.2 and 8q12.3

We tested for the presence of genetic effects independent from the local top hits in 18q12.2 and 8q12.3 (see above). For each of these two SNPs, we first regressed RANlet scores adjusted for population structure against the allelic dosage values and extracted the phenotypic residuals in each dataset. Then we used PLINK v1.9 to test these residuals for association with all the SNPs positioned up to 50 kb from the most significant variant in each region of interest, namely 275 variants on 8q12.3 and 236 variants on 18q12.2. Then we combined the association statistics that were produced for each dataset using METAL (as described above).

SNP×SNP interaction analysis

To investigate potential epistatic effects of rs17663182 and rs16928927 on RANlet, we carried out a two-SNP interaction analysis in R. Since rs16928927 was not available in the Finnish dataset, this analysis was conducted only in the AGS, France, Hungary, Netherlands, and Colorado datasets. The analysis consisted of two steps: first, we regressed RANlet scores adjusted for genetic population structure against the allelic dosages of the SNPs rs17663182 and rs16928927. Then we regressed the RANlet residual scores against a single interaction term of the two SNPs and computed the fraction of phenotypic variance (R^2) explained by this term.

Imaging genetic assessment

To further investigate the potential neurobiological implications of the top association signals detected at rs17663182 (18q12.2) and rs16928927 (8q12.2), we assessed genetic effects of these SNPs on different subcortical volumes, including Nucleus Accumbens, Amygdala, Caudate Nucleus, Hippocampus, Pallidum, Putamen and Thalamus, through the consultation of GWAS summary statistics from a large GWAS meta-analysis on these traits[17]. This study consists of a discovery set of 13,171 subjects of European ancestry, which were made available for consultation by the ENIGMA2 consortium, and of an independent replication dataset of 17,546 individuals. Standardized protocols for image analysis, quality assessment, genetic QC, imputation and association testing were used to ensure data homogeneity within the study[17]. These standardized protocols are openly available online (http://enigma.ini.usc.edu/protocols/). Briefly, the brain measures examined in this study were obtained from structural Magnetic Resonance Imaging (MRI) data collected at participating sites around the world. Brain scans were processed and examined at each site locally, following the standardized protocol procedures. The subcortical brain measures analysed (nucleus accumbens, amygdala, caudate nucleus, hippocampus, pallidum, putamen and thalamus) were delineated in the brain using the segmentation software packages FIRST[18] or FreeSurfer[19]. Similarly, a standardized genotype QC was carried out, on data produced through commercially available platforms (see Table S3 in [17] for details). Further details on scanners used, acquisition protocols, data processing and QC in the different sites can be found in Hibar et al.[17] (see Online Methods and Tables S1-S4). GWAS analyses with the subcortical measures computed as above were conducted at each site, through linear

regression model, except for sites with family data, where a mixed-effects model was used to control for familial relationships. Age, age^2, sex, four MDS components, and intracranial volume were included as covariates. Where applicable, a diagnosis of a neurological/neuropsychiatric disorder, the recruiting centre and the type of scanner used were also included as covariates. The resulting files were combined meta-analytically using a fixed-effect, inverse-variance-weighted model as implemented in METAL[16], both in the discovery and in the replication datasets, and finally a meta-analysis across the discovery and replication data sets was carried out.

Our choice of investigating subcortical brain volumes in relation to our GWAS top hits was determined by two factors, namely i) the increasing evidence implicating subcortical structures in reading and language abilities (as reviewed in [20–22]), and ii) the large sample size of the imaging genetics GWAS[17], which maximized the power to detect significant genetic effects. Previous reviews suggested a potential involvement of some these structures in learning- and language-related abilities, like putamen, thalamus, hippocampus and globus pallidus[20,21], and in reading-related phonological processes[22]. However, since these brain structures are highly interconnected among themselves and with the cortical regions implicated in the above mentioned processes[21], we decided to include in our assessment all the subcortical structures which were tested so far[17].

For this analysis, we computed a Bonferroni-corrected significance threshold $\alpha = 7.1\times10^{-4}$, taking into account two SNPs, five independent latent traits tested in our study (computed in MatSpD, see above), and the seven neuroimaging subcortical regions analysed by Hibar and colleagues[17].

References

1 Landerl K, Ramus F, Moll K, Lyytinen H, Leppänen PHT, Lohvansuu K *et al.* Predictors of developmental dyslexia in European orthographies with varying complexity. *J Child Psychol Psychiatry* 2013; **54**: 686–694.

2 Moll K, Ramus F, Bartling J, Bruder J, Kunze S, Neuhoff N *et al.* Cognitive mechanisms underlying reading and spelling development in five European orthographies. *Learn Instr* 2014; **29**: 65–77.

3 Gialluisi A, Newbury DF, Wilcutt EG, Olson RK, DeFries JC, Brandler WM *et al.* Genome-wide screening for DNA variants associated with reading and language traits. *Genes Brain Behav* 2014; **13**: 686–701.

4 Wechsler D. *The Wechsler intelligence scale for children*. 3rd ed. London: The Psychological Corporation., 1992.

5 Wechsler D. *Wechsler intelligence scale for children.* 4th ed. San Antonio, TX: Psychological Corporation., 2003.

6 Chang CC, Chow CC, Tellier LCAM, Vattikuti S, Purcell SM, Lee JJ. Second-generation PLINK: rising to the challenge of larger and richer datasets. *Gigascience* 2015; **4**: 7.

7 The Genomes Project C. A global reference for human genetic variation. *Nature* 2015; **526**: 68.

8 Delaneau O, Zagury J-F, Marchini J. Improved whole-chromosome phasing for disease and population genetic studies. *Nat Meth* 2013; **10**: 5–6.

9 Howie BN, Donnelly P, Marchini J. A flexible and accurate genotype imputation method for the next generation of genome-wide association studies. *PLoS Genet* 2009; **5**: e1000529.

10 Li J, Ji L. Adjusting multiple testing in multilocus analyses using the eigenvalues of a correlation matrix. *Heredity (Edinb)* 2005; **95**: 221–227.

11 Purcell S, Cherny SS, Sham PC. Genetic Power Calculator: design of linkage and association genetic mapping studies of complex traits. *Bioinformatics* 2003; **19**: 149–150.

12 Aulchenko YS, Ripke S, Isaacs A, van Duijn CM. GenABEL: an R library for genome-wide association analysis. *Bioinformatics* 2007; **23**: 1294–1296.

13 R Core Team. *R: A Language and Environment for Statistical Computing*. R Foundation for Statistical Computing: Vienna, Austria., 2015.

14 Langfelder P, Horvath S. WGCNA: an R package for weighted correlation network analysis. *BMC Bioinformatics* 2008; **9**: 559.

15 van der Sluis S, Posthuma D, Dolan C V. TATES: Efficient Multivariate Genotype-Phenotype Analysis for Genome-Wide Association Studies. *PLOS Genet* 2013; **9**: e1003235.

16 Willer CJ, Li Y, Abecasis GR. METAL: fast and efficient meta-analysis of genomewide association scans. *Bioinformatics* 2010; **26**: 2190–2191.

17 Hibar DP, Stein JL, Renteria ME, Arias-Vasquez A, Desrivières S, Jahanshad N *et al.* Common genetic variants influence human subcortical brain structures. *Nature* 2015; **520**: 224–229.

18 Patenaude B, Smith SM, Kennedy DN, Jenkinson M. A Bayesian model of shape and appearance for subcortical brain segmentation. *Neuroimage* 2011; **56**: 907–922.

19 Fischl B, Salat DH, Busa E, Albert M, Dieterich M, Haselgrove C *et al.* Whole Brain Segmentation: Automated Labeling of Neuroanatomical Structures in the Human Brain. *Neuron* 2002; **33**: 341–355.

20 Eicher JD, Gruen JR. Imaging-genetics in dyslexia: Connecting risk genetic variants to brain neuroimaging and ultimately to reading impairments. *Mol Genet Metab* 2013; **110**: 201–212.

21 Krishnan S, Watkins KE, Bishop DVM. Neurobiological Basis of Language Learning Difficulties. *Trends Cogn Sci* 2016; **20**: 701–714.

22 Mascheretti S, De Luca A, Trezzi V, Peruzzo D, Nordio A, Marino C *et al.* Neurogenetics of developmental dyslexia: From genes to behavior through brain neuroimaging and cognitive and sensorial mechanisms. *Transl Psychiatry* 2017; **7**: e987-15.

URLs

PLINK v1.9: https://www.cog-genomics.org/plink2

QCTOOL v1.4: http://www.well.ox.ac.uk/~gav/qctool/

Genetic Power Calculator: http://zzz.bwh.harvard.edu/gpc/

GenABEL: http://www.genabel.org/

R v3.2.3: http://www.R-project.org/

METAL v25-03-2011: http://www.sph.umich.edu/csg/abecasis/Metal/index.html
