# Supplementary material for: Genome-wide association scan identifies new variants associated with a cognitive predictor of dyslexia
Source: Transl Psychiatry. 2019 Feb 11;9:77. doi: 10.1038/s41398-019-0402-0 (PMC6370792; doi:10.1038/s41398-019-0402-0)
Supplement: Supplementary file 2 — Supplementary Results: Manhattan and QQ plots [file 41398_2019_402_MOESM2_ESM.docx]

**Supplementary Results: Manhattan and QQ plots**

a)


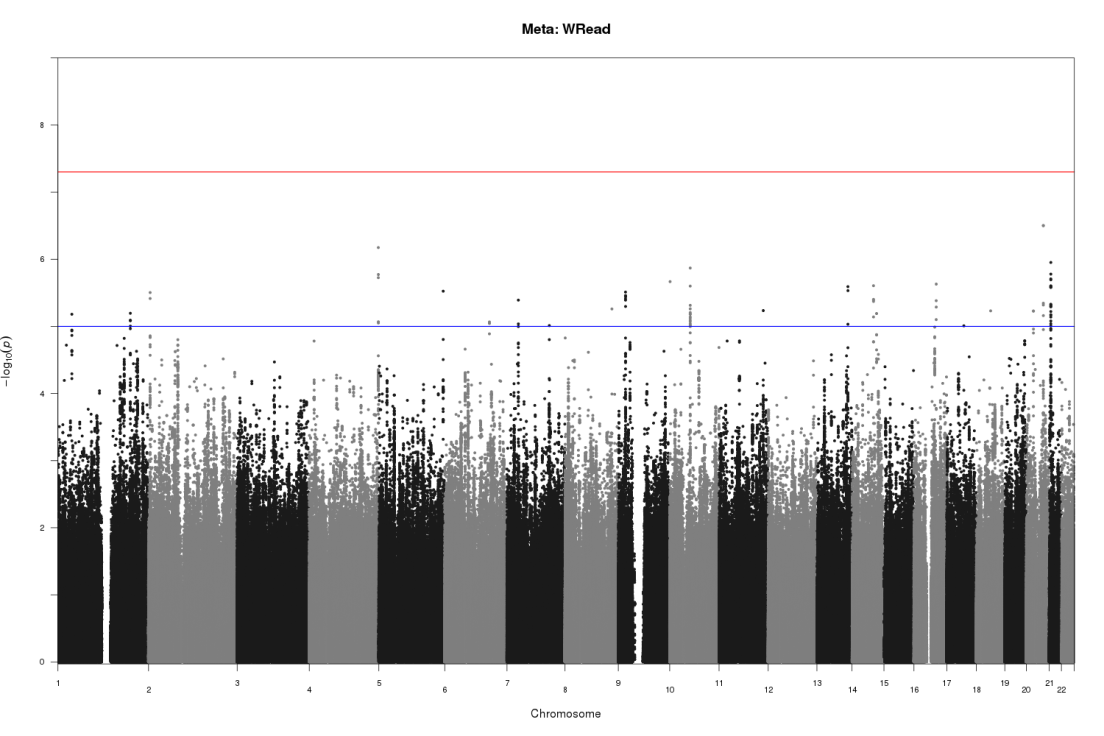


b)


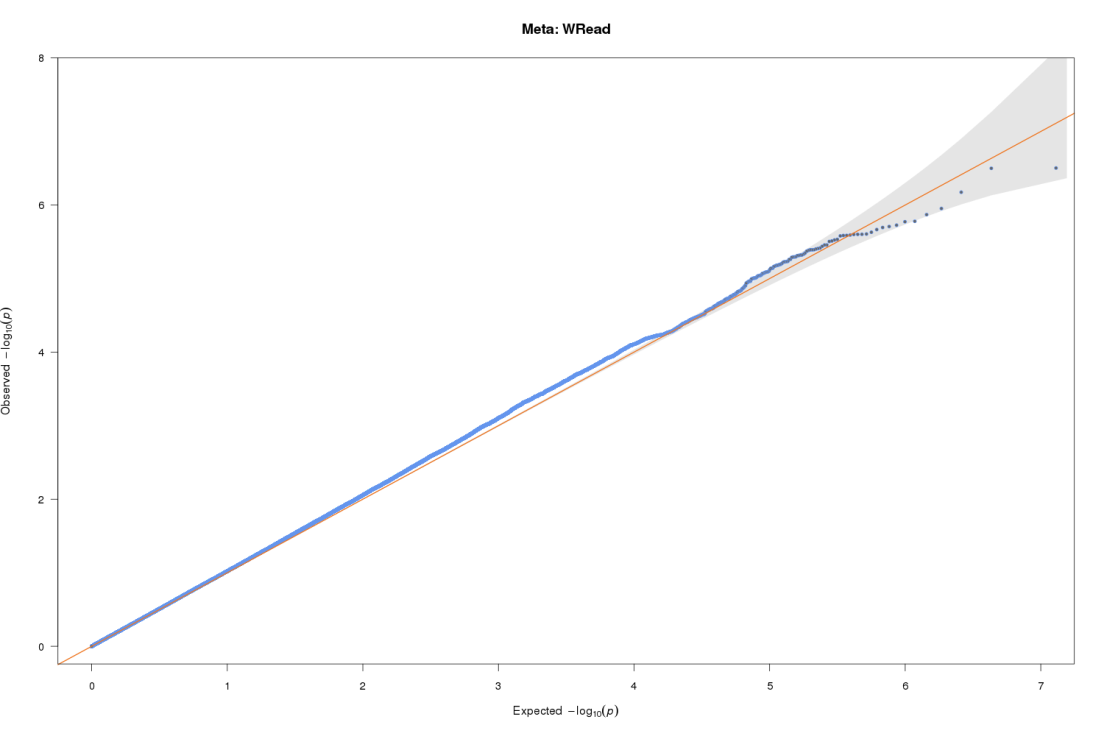


c)


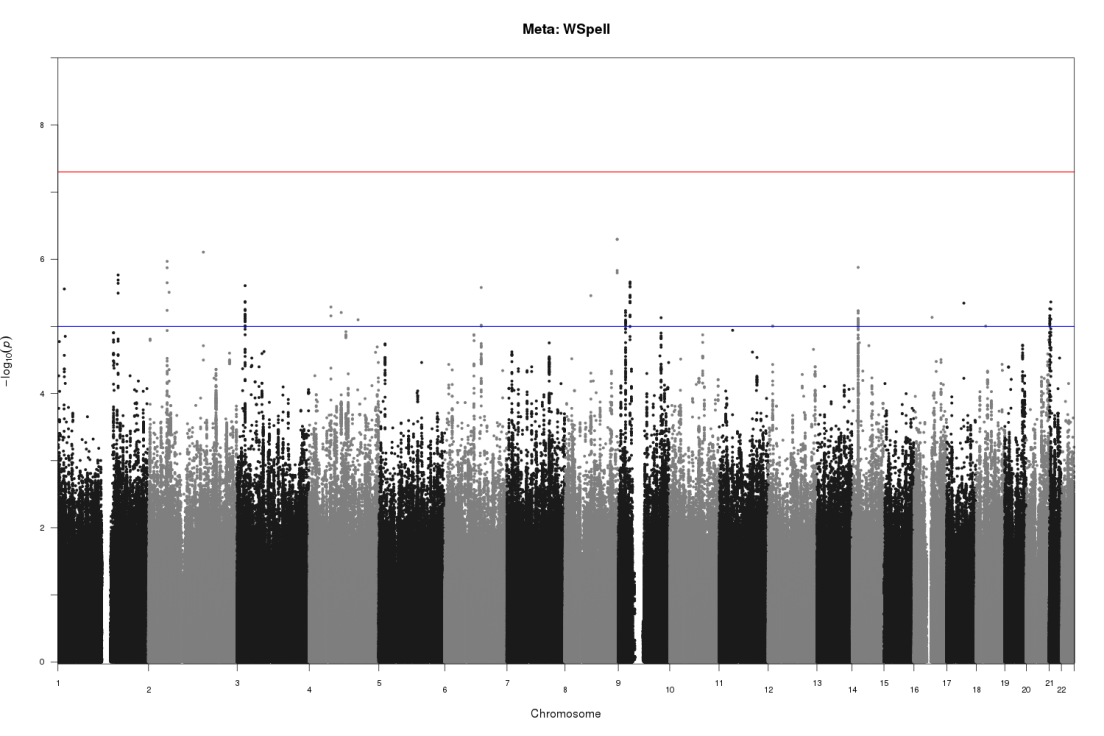


d)


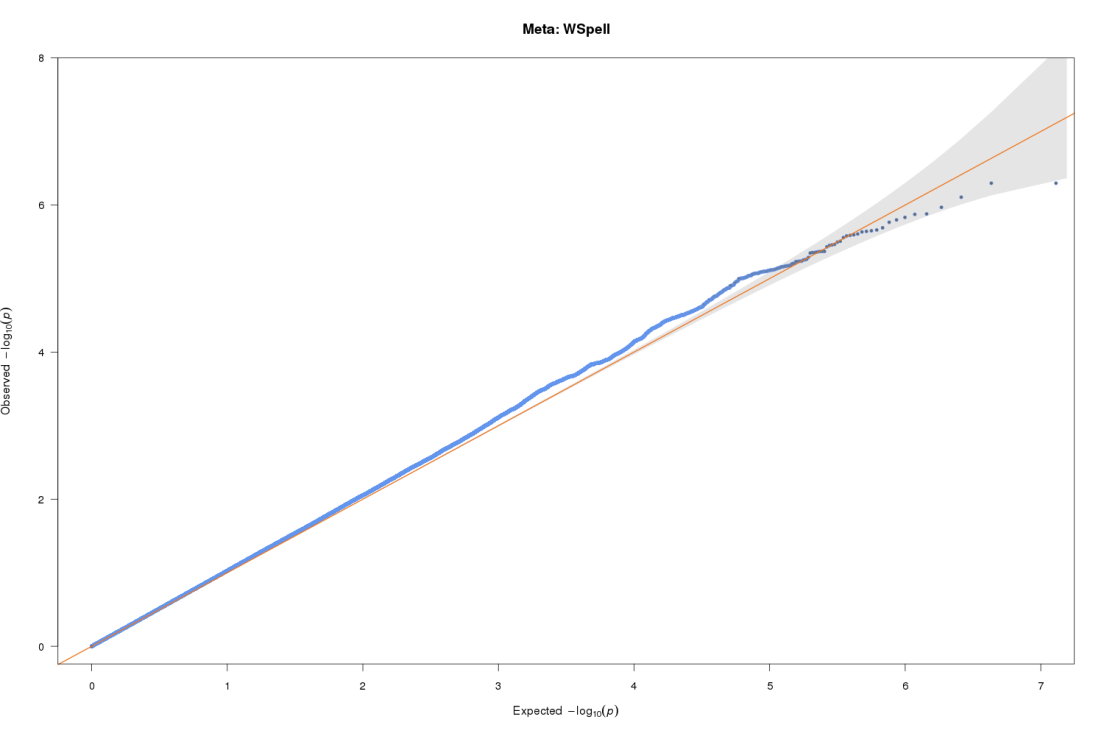


e)


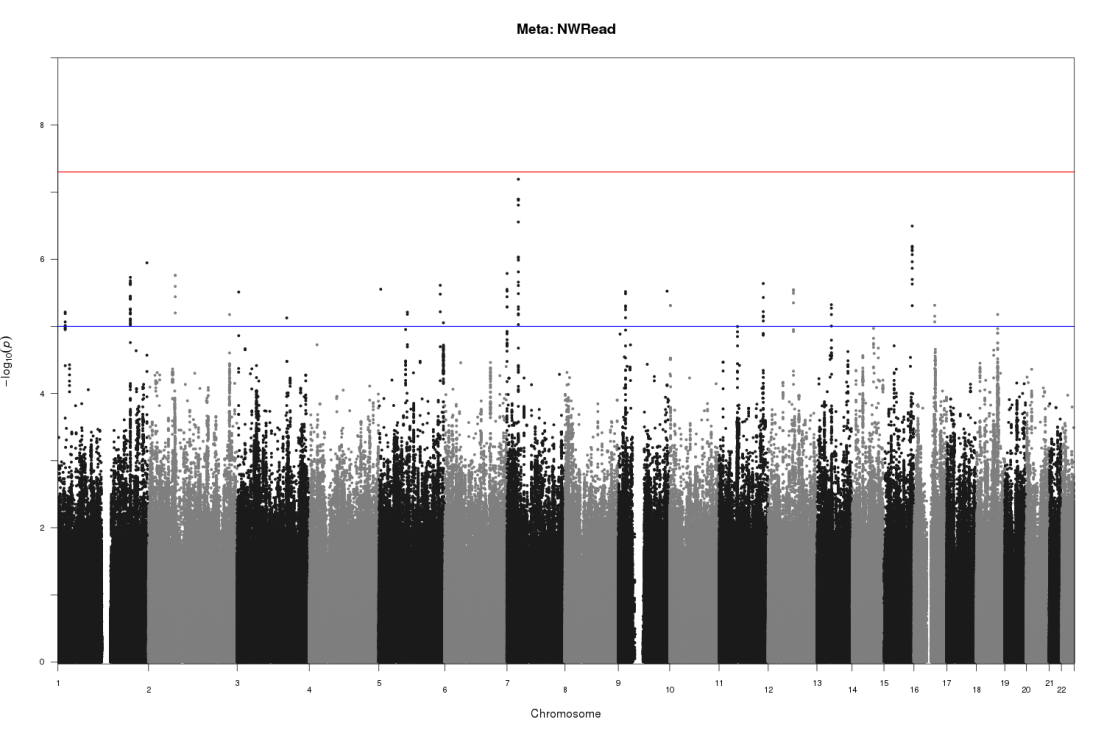


f)


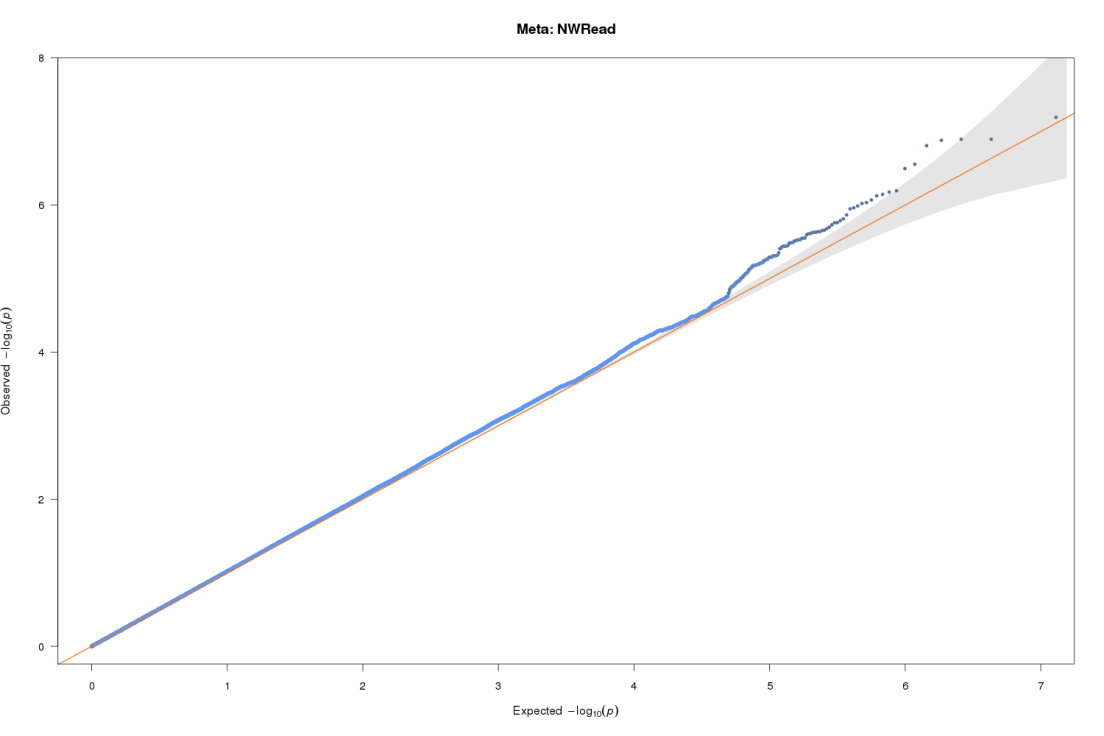


g)


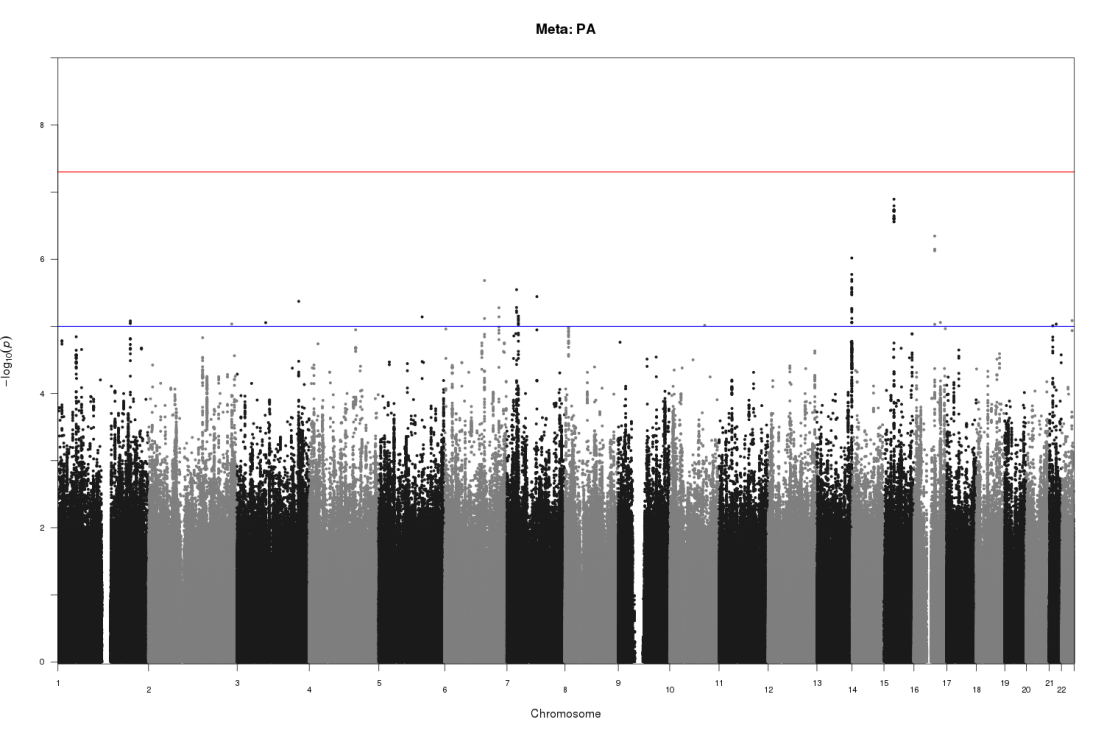


h)


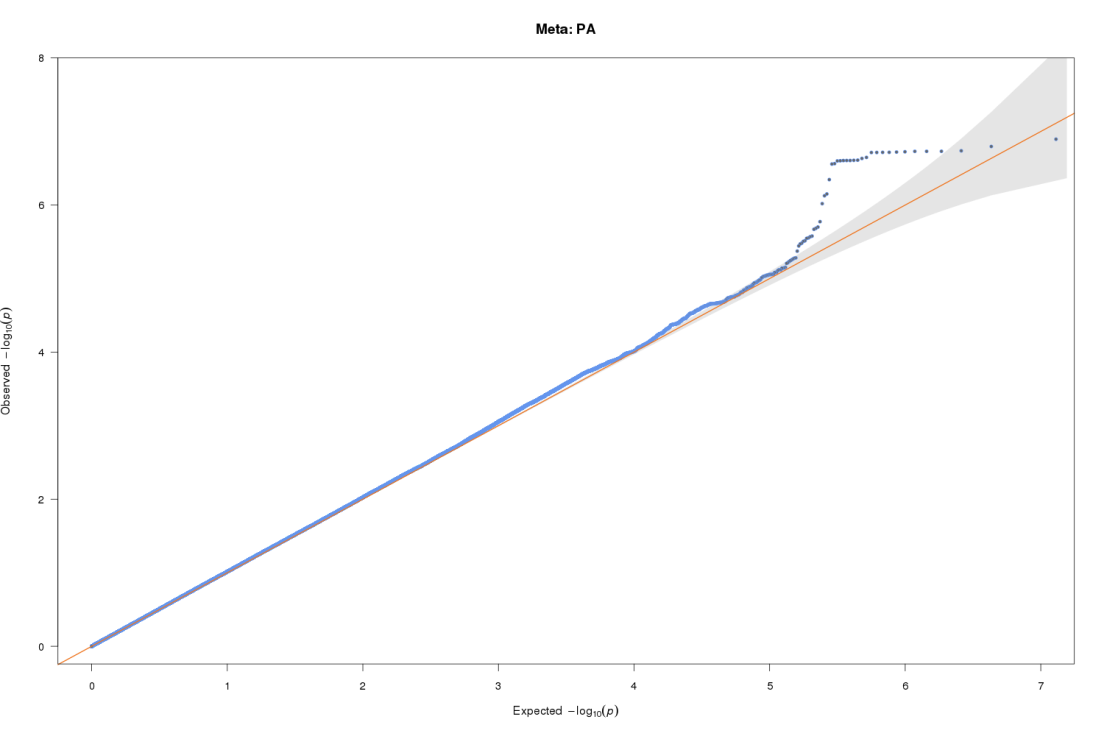


i)


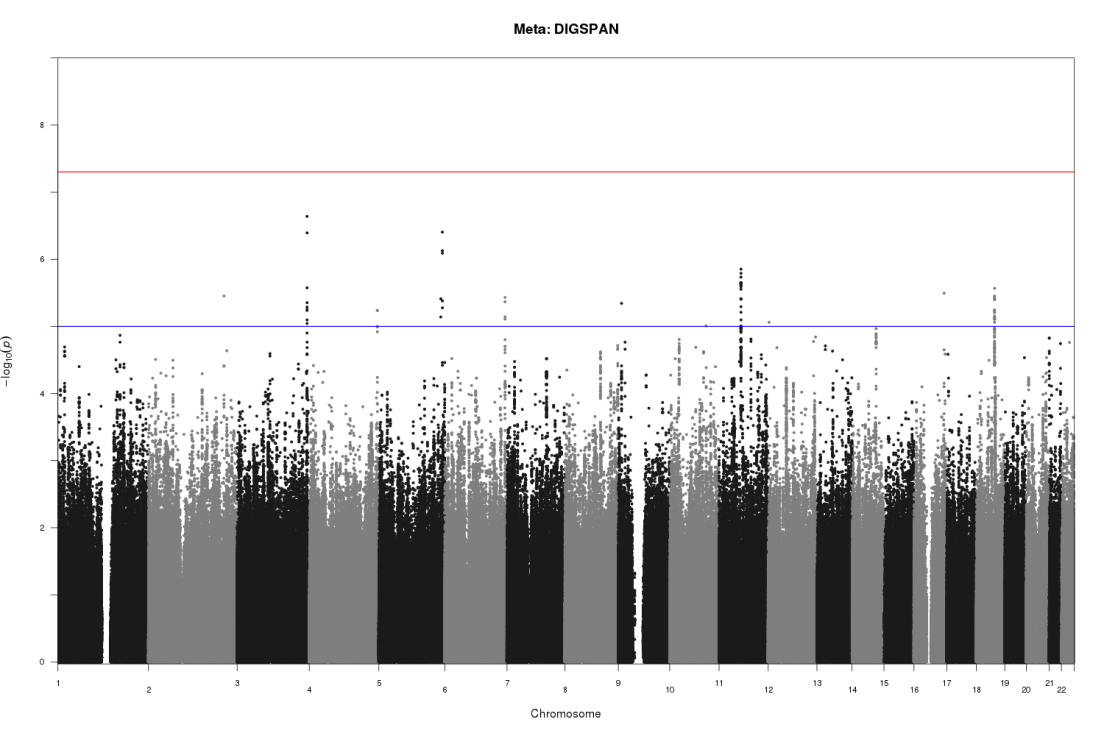


j)


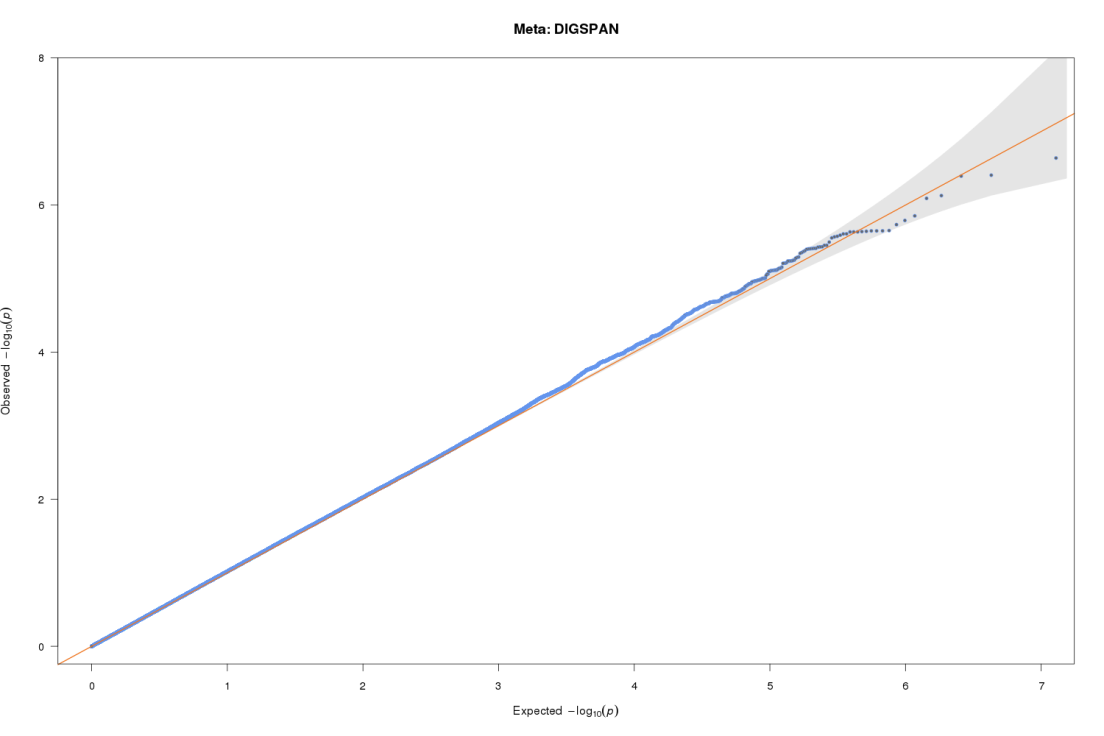


k)


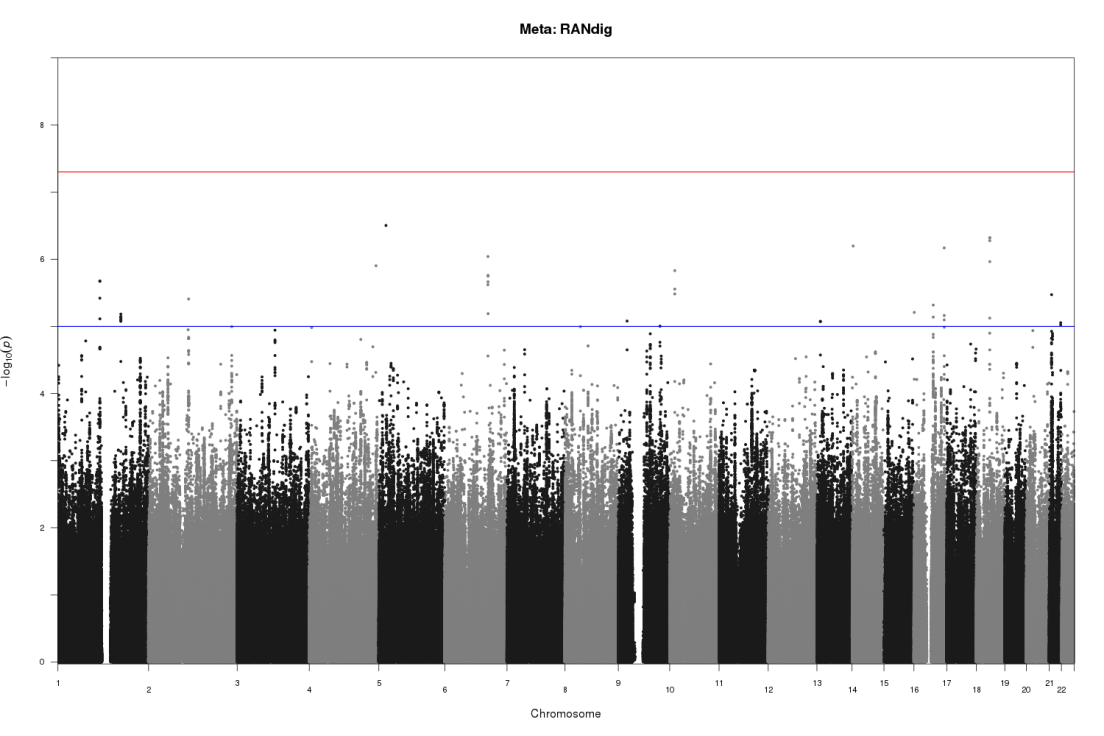


l)


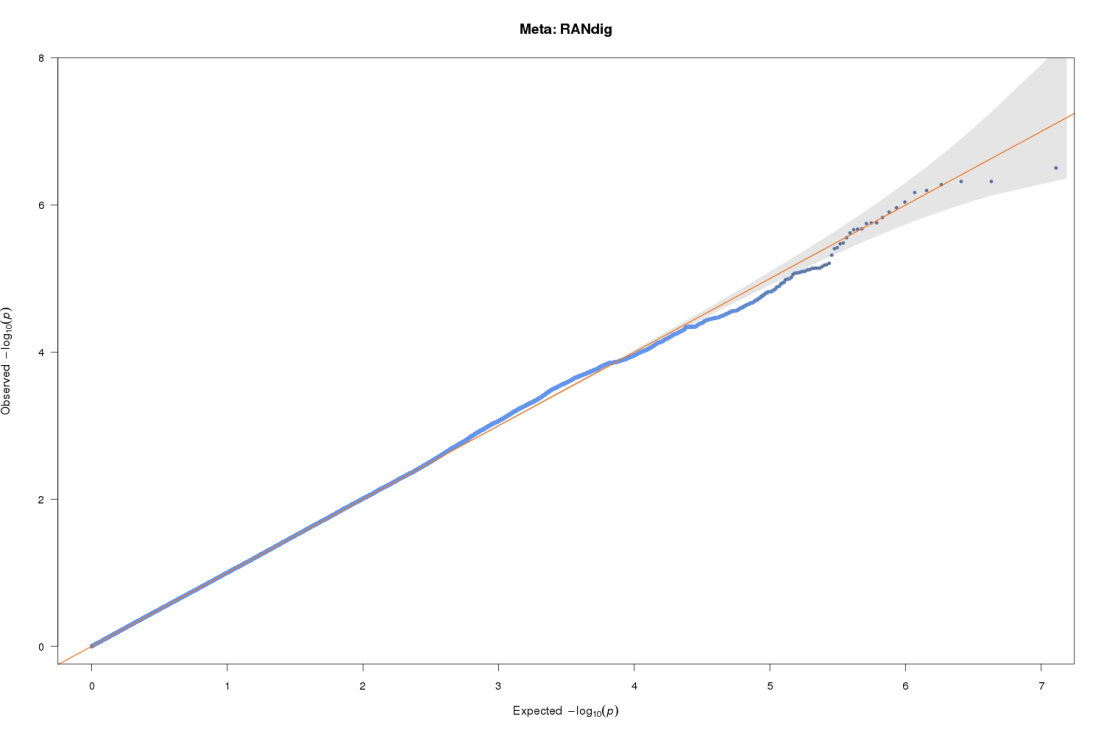


m)


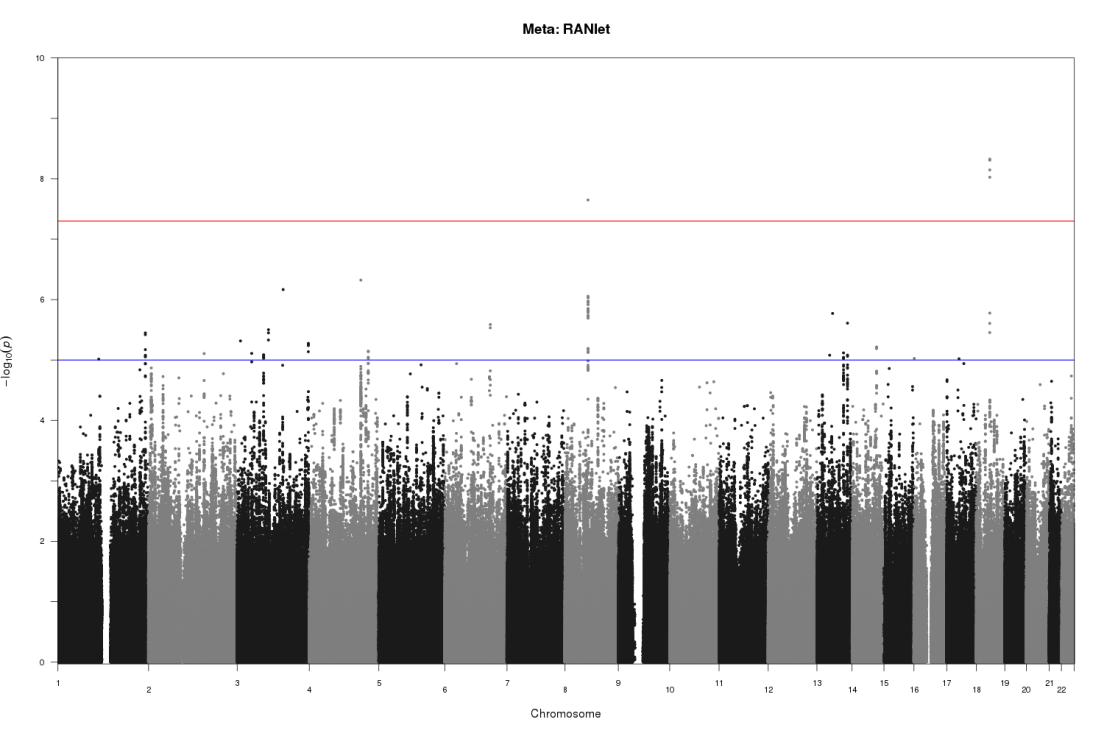


n)


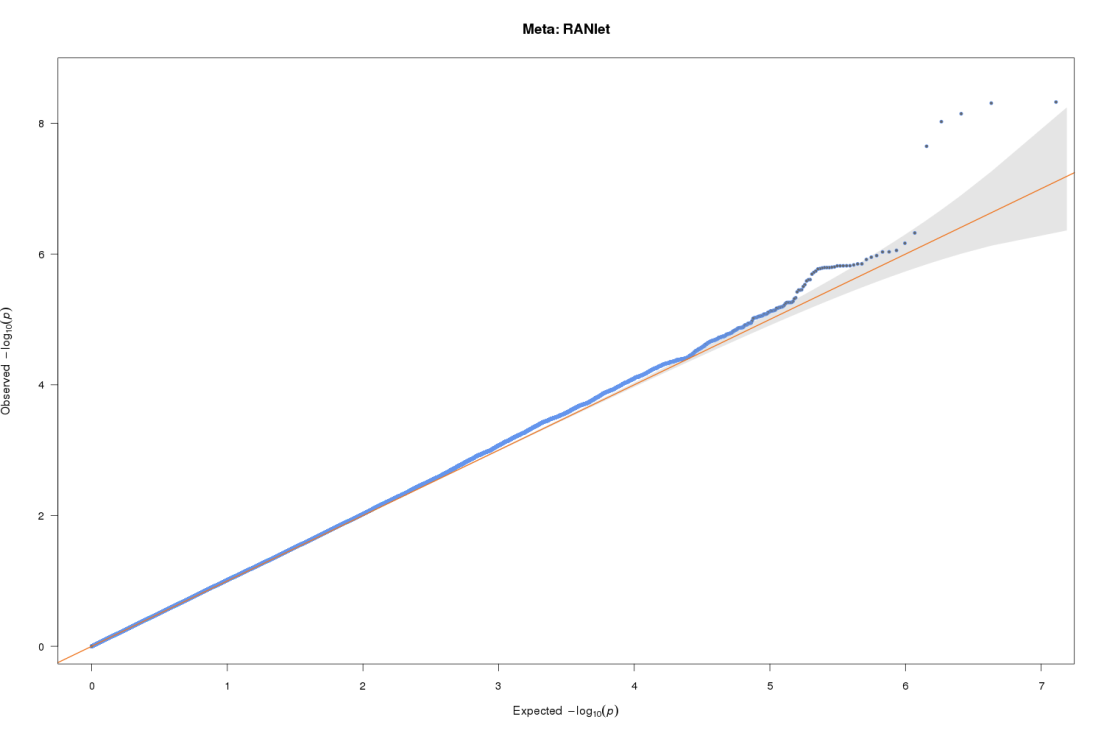


o)


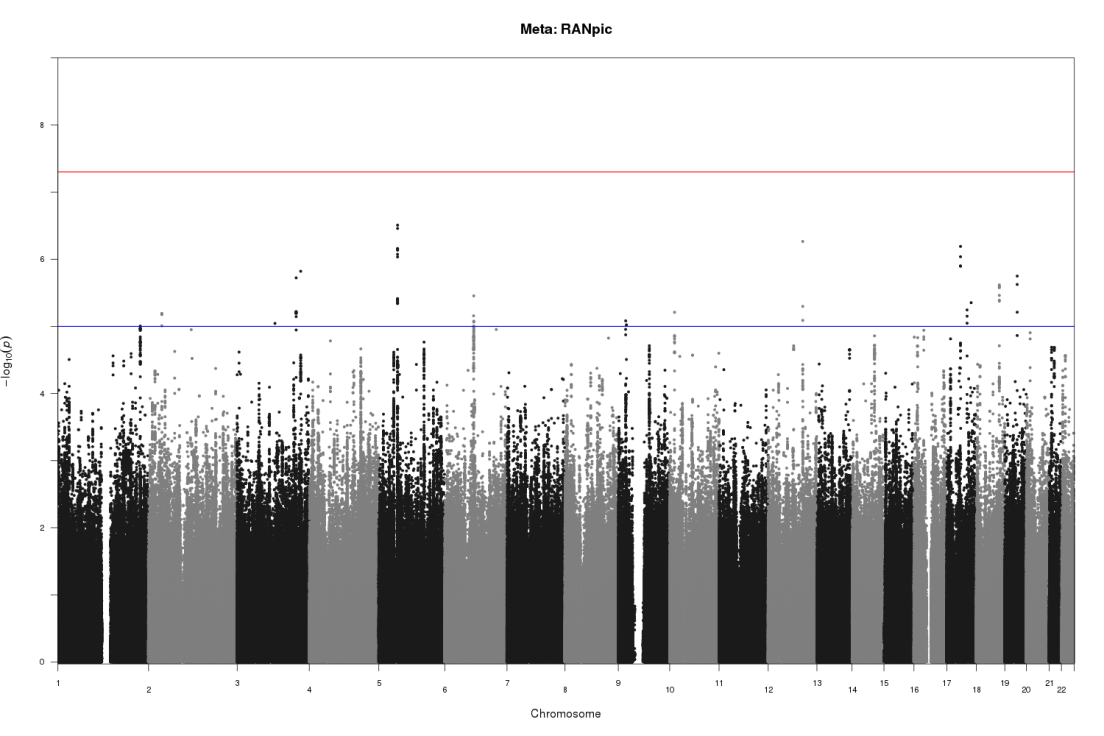


p)


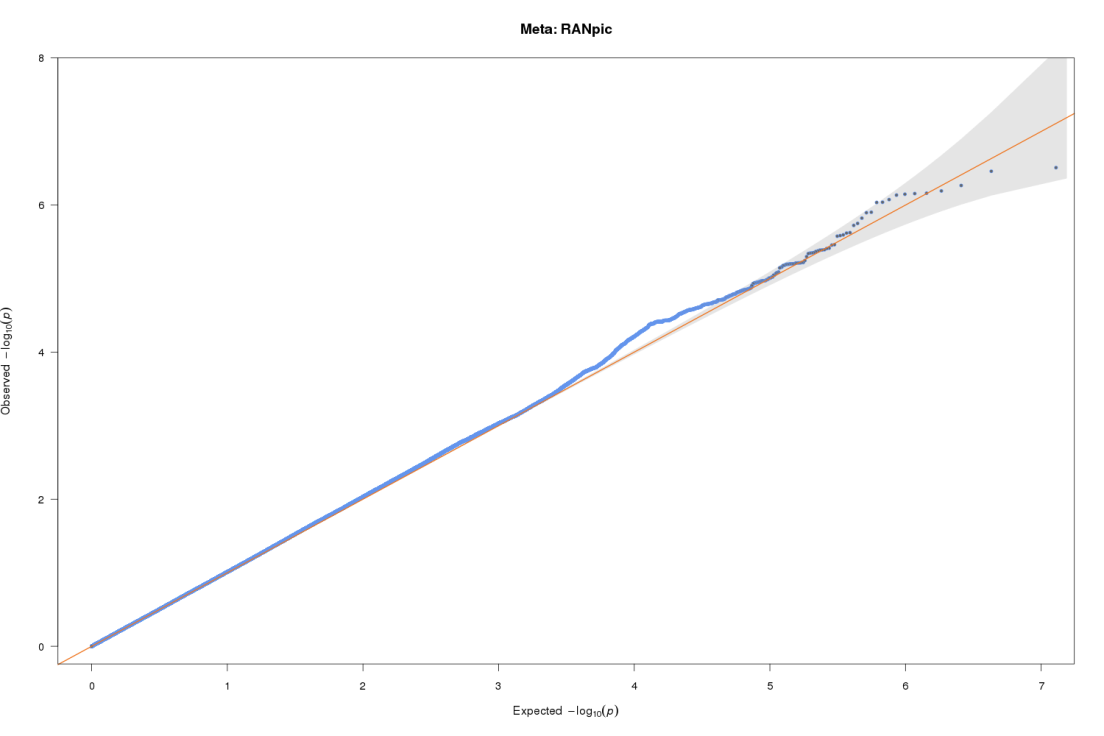


**Figure S1.** Manhattan and QQ plots of the GWAS analyses carried out in the present study. **a-b)** Word Reading (WRead); **c-d)** Word Spelling (WSpell); **e-f)** Nonword Reading (NWRead); **g-h)** Phoneme Awareness (PA); **i-j)** Digit Span (DigSpan); Rapid Automatized Naming of **k-l)** digits (RANdig); **m-n)** letters (RANlet); and **o-p)** pictures (RANpic).
