# Supplementary material for: Genome-wide association scan identifies new variants associated with a cognitive predictor of dyslexia
Source: Transl Psychiatry. 2019 Feb 11;9:77. doi: 10.1038/s41398-019-0402-0 (PMC6370792; doi:10.1038/s41398-019-0402-0)
Supplement: Supplementary file 3 — Supplementary Results: Characterization of top association signals [file 41398_2019_402_MOESM3_ESM.docx]

**Supplementary Results: Characterization of top association signals**

**a)**

| **Dataset** | **A1** | **A2** | **A1 Freq** | **Beta ^a^** | **Beta SE** | **P** | **Effect size ^b^** | **Empirical P ^c^** |
| --- | --- | --- | --- | --- | --- | --- | --- | --- |
| AGS | G | T | 0.9152 | 0.41 | 0.09 | 6.5**×**10^-6^ | 0.018 | 6.84**×**10^-6^ |
| Finland | G | T | 0.9448 | 0.419 | 0.205 | 0.042 | 0.013 | 0.043 |
| France | G | T | 0.9287 | 0.446 | 0.314 | 0.159 | 0.017 | 0.156 |
| Netherlands | G | T | 0.9291 | 0.064 | 0.238 | 0.787 | 3.3**×**10^-4^ | 0.787 |
| Hungary | G | T | 0.8996 | 0.294 | 0.188 | 0.119 | 0.01 | 0.12 |
| Colorado | G | T | 0.9082 | 0.317 | 0.117 | 0.007 | 0.012 | 0.011 |

**b)**

| **Dataset** | **A1** | **A2** | **A1 Freq** | **Beta ^a^** | **Beta SE** | **P** | **Effect size ^b^** | **Empirical P ^c^** |
| --- | --- | --- | --- | --- | --- | --- | --- | --- |
| AGS | C | T | 0.9384 | -0.286 | 0.104 | 0.006 | 0.007 | 0.006 |
| France | C | T | 0.9146 | -0.512 | 0.306 | 0.097 | 0.023 | 0.098 |
| Netherlands | C | T | 0.9418 | -0.482 | 0.262 | 0.067 | 0.015 | 0.064 |
| Hungary | C | T | 0.9405 | -0.569 | 0.216 | 0.009 | 0.03 | 0.008 |
| Colorado | C | T | 0.946 | -0.495 | 0.137 | 3.2**×**10^-4^ | 0.022 | 6.4**×**10^-4^ |

**Tables S5.** Detailed association statistics by dataset of the SNPs **a)** rs17663182 and **b)** rs16928927 with the RANlet score. Note: rs16928927 was not available for association testing in Finland.

^a^ Beta values refer to A1.

^b^ Proportion of RANlet variance explained by each SNP, computed as linear regression R^2^ of the trait vs SNP allelic dosage. Caution is suggested in the interpretation of these data since they may be inflated due to the winner curse effect^1^.

^c^ Permutation-based correlation test between allelic dosage of each SNP and RANlet score.

**c)**

| **Trait** | **N** | **Zscore** | **P-value** | **Direction^a^** | **HetISq ^b^** | **HetPVal ^b^** |
| --- | --- | --- | --- | --- | --- | --- |
| WRead | 2595 | 0.84 | 0.401 | +++-+- | 63.5 | 0.018 |
| WSpell | 2547 | -0.481 | 0.63 | +++-+- | 0 | 0.661 |
| NWRead | 2541 | 0.766 | 0.444 | +++-+- | 55.5 | 0.047 |
| PA | 2555 | -0.579 | 0.563 | -++++- | 24.9 | 0.248 |
| DigSpan | 2591 | 0.158 | 0.874 | -++++- | 44.6 | 0.108 |
| RANdig | 2563 | 1.429 | 0.153 | +-++++ | 0 | 0.824 |
| RANpic | 2562 | 0.905 | 0.366 | ++-+-- | 0 | 0.69 |

**d)**

| **Trait** | **N** | **Zscore** | **P-value** | **Direction^a^** | **HetISq ^b^** | **HetPVal ^b^** |
| --- | --- | --- | --- | --- | --- | --- |
| WRead | 2271 | -0.152 | 0.879 | +-++- | 0 | 0.569 |
| WSpell | 2227 | 0.59 | 0.556 | +-++- | 0 | 0.965 |
| NWRead | 2241 | -0.42 | 0.675 | -+-+- | 0 | 0.547 |
| PA | 2231 | -0.27 | 0.787 | --++- | 0 | 0.732 |
| DigSpan | 2267 | -0.25 | 0.802 | -+-++ | 2 | 0.395 |
| RANdig | 2240 | -0.121 | 0.904 | -+++- | 37.4 | 0.172 |
| RANpic | 2239 | 0.336 | 0.737 | -+-++ | 0 | 0.752 |

**Tables S5.** Test of pleiotropy for the SNPs **c)** rs17663182 and **d)** rs16928927. The results of the association tests in the different datasets were meta-analysed in METAL^2^, to check the concordance of allelic trends across datasets. Note: uncorrected association statistics are reported.

^a^ The direction of effect of the major alleles (G and C, respectively) is reported for datasets in the following order: **c)** AGS, Finland, France, Netherlands, Hungary and Colorado; **d)** AGS, France, Netherlands, Hungary and Colorado.

^b^ I-squared test for heterogeneity of effects across datasets and relevant p-value.

**e)**

| **Variant** | **A1** | **A2** | **N** | **Zscore** | **P-value** | **Direction^a^** | **HetISq ^b^** | **HetPVal ^b^** |
| --- | --- | --- | --- | --- | --- | --- | --- | --- |
| rs117685376 | T | G | 548 | 2.761 | 0.006 | ?+?+?? | 0 | 0.918 |
| rs200051549 | T | TG | 225 | 1.955 | 0.051 | ???+?? | 0 | 1 |
| rs72890217 | T | C | 2563 | -1.482 | 0.138 | ------ | 0 | 0.866 |
| rs55726734 | A | T | 2563 | -1.477 | 0.14 | ------ | 0 | 0.798 |
| rs8090547 | T | C | 2563 | -1.25 | 0.211 | ---+-- | 0 | 0.925 |
| rs56058427 | T | C | 2327 | -1.218 | 0.223 | --+-?+ | 26.2 | 0.247 |
| rs79422859 | T | C | 1081 | -1.215 | 0.225 | ?+?-?- | 4.5 | 0.351 |
| rs77071558 | T | G | 1081 | -1.214 | 0.225 | ?+?-?- | 3.5 | 0.355 |
| rs78087751 | T | G | 1201 | -1.201 | 0.23 | ?-+-?- | 72.8 | 0.012 |
| rs143147075 | T | C | 1201 | 1.061 | 0.289 | ?-++?+ | 0 | 0.677 |

**f)**

| **Variant** | **A1** | **A2** | **N** | **Zscore** | **P-value** | **Direction^a^** | **HetISq ^b^** | **HetPVal ^b^** |
| --- | --- | --- | --- | --- | --- | --- | --- | --- |
| rs72649395 | A | C | 2240 | 1.91 | 0.056 | +++++ | 0 | 1.0 |
| rs6982729 | A | C | 2240 | -1.845 | 0.065 | ----- | 0.6 | 0.403 |
| rs7835925 | A | G | 2240 | -1.621 | 0.105 | ++--- | 60.7 | 0.037 |
| rs55737832 | A | G | 2240 | 1.32 | 0.187 | +-+++ | 0 | 0.582 |
| rs78905768 | A | G | 2015 | -1.088 | 0.277 | --?+- | 54 | 0.089 |
| rs35113272 | A | G | 2240 | -1.026 | 0.305 | --++- | 0 | 0.655 |
| rs62511508 | A | G | 2240 | 1.02 | 0.308 | --+++ | 29.7 | 0.223 |
| rs1993125 | A | G | 2240 | -1.016 | 0.31 | ++--- | 29.7 | 0.224 |
| rs7001218 | A | C | 2240 | 1.015 | 0.31 | --+++ | 26.3 | 0.246 |
| rs7000955 | A | G | 2240 | 1.014 | 0.31 | --+++ | 30.6 | 0.218 |

**Tables S5.** Test for independent genetic effects in **e)** 18q12.2 and **f)** 8q12.3. The most significant associations independent from **e)** rs17663182 and **f)** rs16928927, within 50 kb from the local top hit, are reported. The results of the association tests in the different datasets were meta-analysed in METAL^2^, to check the concordance of allelic trends across datasets. Note: uncorrected association statistics are reported, which need to be adjusted for the number of variants tested within each region (275 variants on 8q12.3 and 236 variants on 18q12.2).

^a^ The direction of effect is reported for datasets in the following order: **e)** AGS, Finland, France, Netherlands, Hungary and Colorado; **f)** AGS, France, Netherlands, Hungary and Colorado.

^b^ I-squared test for heterogeneity of effects across datasets and relevant p-value.

**g)**

| **Dataset** | **AGS** | **France** | **Hungary** | **Netherlands** | **Colorado** |
| --- | --- | --- | --- | --- | --- |
| R^2^ | 5.7**×**10^-6^ | 7.9**×**10^-4^ | 4.9**×**10^-4^ | 0.006 | 0.003 |
| P | 1.0 | 0.993 | 0.99 | 0.7 | 0.661 |

**Table S5g.** SNP-SNP interaction analysis of rs17663182 (18q12.2) and rs16928927 (8q12.3) on RANlet. Regression R^2^ of the interaction term and relevant p-values are presented here by single dataset. Since rs16928927 was not available in the Finnish dataset, this analysis was conducted only in the AGS, France, Hungary, Netherlands and Colorado datasets.

**h)**

| **Subcortical structure** | **Effect**  **Allele** | **Non-Effect**  **Allele** | **Freq 1000G**  **EUR** | **Beta** | **StdErr** | **Pvalue** | **N** |
| --- | --- | --- | --- | --- | --- | --- | --- |
| hippocampus | T | G | 0.0686 | 7.448 | 8.47 | 0.379 | 13163 |
| accumbens | T | G | 0.0686 | 3.134 | 1.971 | 0.112 | 13112 |
| amygdala | T | G | 0.0686 | 0.696 | 4.255 | 0.87 | 13160 |
| caudate | T | G | 0.0686 | 18.522 | 8.799 | 0.035 | 13171 |
| pallidum | T | G | 0.0686 | 5.996 | 3.447 | 0.082 | 13142 |
| putamen | T | G | 0.0686 | 14.303 | 10.756 | 0.184 | 13145 |
| thalamus | T | G | 0.0686 | 13.344 | 11.266 | 0.236 | 13193 |

**i)**

| **Subcortical structure** | **Effect**  **Allele** | **Non-Effect**  **Allele** | **Freq 1000G**  **EUR** | **Beta** | **StdErr** | **Pvalue** | **N** |
| --- | --- | --- | --- | --- | --- | --- | --- |
| hippocampus | T | C | 0.0501 | -0.582 | 9.653 | 0.952 | 11883 |
| accumbens | T | C | 0.0501 | 1.467 | 2.292 | 0.522 | 11832 |
| amygdala | T | C | 0.0501 | 4.798 | 4.861 | 0.324 | 11880 |
| caudate | T | C | 0.0501 | 14.508 | 10.109 | 0.151 | 11891 |
| pallidum | T | C | 0.0501 | 10.736 | 3.944 | 0.006 | 11862 |
| putamen | T | C | 0.0501 | 5.738 | 12.314 | 0.641 | 11865 |
| thalamus | T | C | 0.0501 | 14.98 | 12.784 | 0.241 | 11913 |

**Tables S5.** Association of the SNPs **h)** rs17663182 and **i)** rs16928927 with seven subcortical volumes tested by Hibar and colleagues^3^.

**a)**


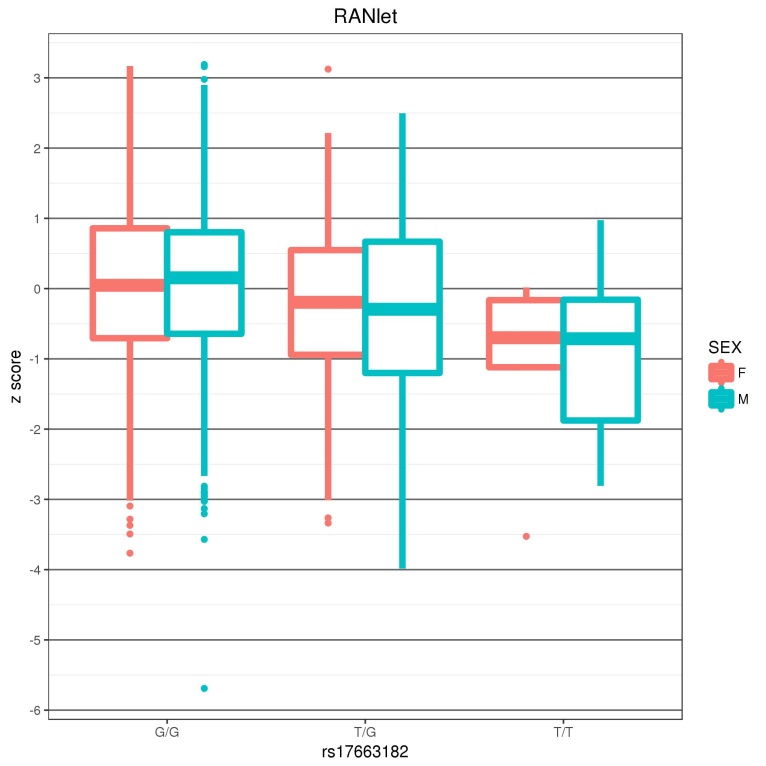


**b)**


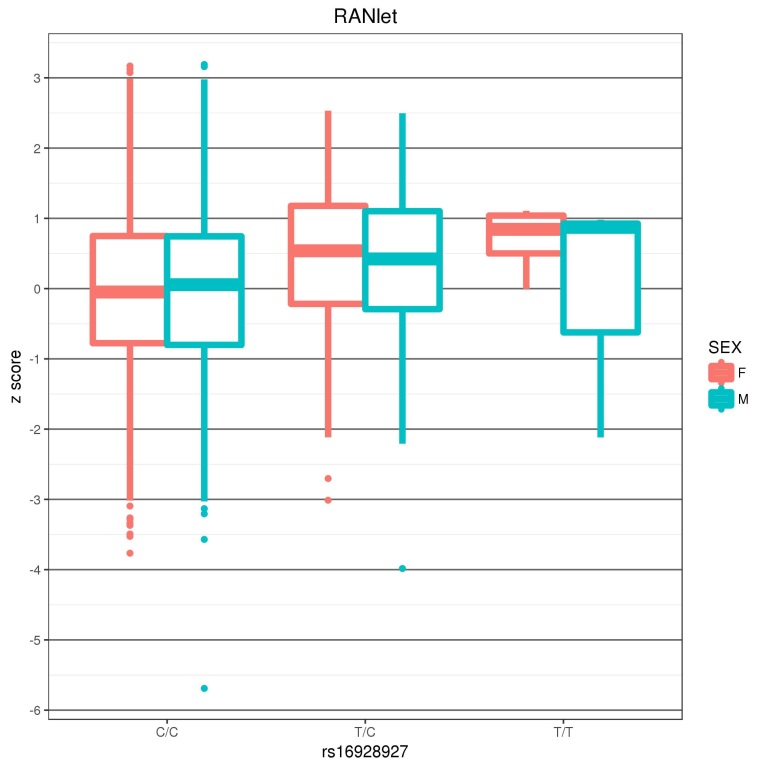


**Figure S5.** Boxplots of the RANlet score as a function of genotype of the lead variants **a)** rs17663182 (major allele G) and **b)** rs16928927 (major allele C). To generate these plots, all datasets were pooled together. RANlet Z-scores plotted here are residualized against the first 10 MDS covariates in all datasets except for Colorado, where we adjusted the phenotypic measure for pairwise genetic relatedness in GenABEL (see *Supplementary Methods* section).

**References**

1 Ioannidis JPA. Why Most Discovered True Associations Are Inflated. *Epidemiology* 2008; **19**: 640–648.

2 Willer CJ, Li Y, Abecasis GR. METAL: fast and efficient meta-analysis of genomewide association scans. *Bioinformatics* 2010; **26**: 2190–2191.

3 Hibar DP, Stein JL, Renteria ME, Arias-Vasquez A, Desrivières S, Jahanshad N *et al.* Common genetic variants influence human subcortical brain structures. *Nature* 2015; **520**: 224–229.
