# Supplementary material for: Genome-wide association scan identifies new variants associated with a cognitive predictor of dyslexia
Source: Transl Psychiatry. 2019 Feb 11;9:77. doi: 10.1038/s41398-019-0402-0 (PMC6370792; doi:10.1038/s41398-019-0402-0)
Supplement: Supplementary file 9 — Supplementary Results: PRS analysis [file 41398_2019_402_MOESM9_ESM.docx]

**Supplementary Results: Polygenic Risk Score analysis**

a)

| Trait | WRead | WSpell | NWRead | PA | DigSpan | RANdig | RANlet | RANpic |
| --- | --- | --- | --- | --- | --- | --- | --- | --- |
| ADHD | **6.8×10^-3^** | **6.1×10^-3^** | **4.3×10^-3^** | 1.7×10^-3^ | 5.4×10^-3^ | 1.8×10^-5^ | 2.4×10^-4^ | 7.4×10^-5^ |
| ASD | 1.2×10^-7^ | 3.5×10^-4^ | 6.6×10^-4^ | 4.2×10^-5^ | 1.4×10^-4^ | 3.6×10^-6^ | 8.2×10^-5^ | 4.3×10^-5^ |
| MDD | 1.0×10^-4^ | 1.6×10^-4^ | 2.0×10^-4^ | 6.2×10^-4^ | 1.2×10^-3^ | 1.0×10^-4^ | 9.6×10^-6^ | 2.1×10^-6^ |
| SCZ | 2.1×10^-5^ | 3.2×10^-4^ | 1.8×10^-3^ | 1.2×10^-4^ | 1.1×10^-4^ | 8.7×10^-4^ | 1.7×10^-4^ | 4.4×10^-4^ |
| EDUyears | **0.019** | **0.013** | **0.013** | **0.019** | **0.011** | 7.7×10^-5^ | 1.4×10^-3^ | 1.5×10^-3^ |
| Accumbens | 6.3×10^-5^ | 9.8×10^-5^ | 1.5×10^-8^ | 5.1×10^-5^ | 5.5×10^-4^ | 3.0×10^-6^ | 1.5×10^-4^ | 4.0×10^-4^ |
| Amygdala | 1.3×10^-4^ | 2.1×10^-4^ | 1.7×10^-5^ | 3.8×10^-4^ | 2.1×10^-3^ | 3.3×10^-4^ | 2.1×10^-4^ | 4.9×10^-4^ |
| Caudate | 3.1×10^-5^ | 2.2×10^-4^ | 4.4×10^-5^ | 9.0×10^-4^ | 9.8×10^-4^ | 2.8×10^-5^ | 4.7×10^-4^ | 4.4×10^-4^ |
| Hippocampus | 1.8×10^-4^ | 7.6×10^-5^ | 1.0×10^-5^ | 8.1×10^-4^ | 1.4×10^-4^ | 2.0×10^-4^ | 1.3×10^-5^ | 1.3×10^-3^ |
| Pallidum | 2.5×10^-4^ | 1.7×10^-5^ | 3.1×10^-4^ | 2.0×10^-4^ | 1.7×10^-4^ | 2.8×10^-4^ | 6.5×10^-4^ | 7.9×10^-6^ |
| Putamen | 2.4×10^-4^ | 6.0×10^-4^ | 9.2×10^-5^ | 2.6×10^-3^ | 2.5×10^-4^ | 2.3×10^-4^ | 1.1×10^-3^ | 4.2×10^-6^ |
| Thalamus | 1.7×10^-4^ | 1.0×10^-3^ | 1.9×10^-3^ | 2.2×10^-3^ | 2.7×10^-3^ | 5.3×10^-6^ | 5.5×10^-5^ | 6.6×10^-4^ |

b)

| Trait | WRead | WSpell | NWRead | PA | DigSpan | RANdig | RANlet | RANpic |
| --- | --- | --- | --- | --- | --- | --- | --- | --- |
| ADHD | **5.99×10^-7^** | **2.52×10^-6^** | **6.68×10^-5^** | 0.012 | 8.55×10^-5^ | 0.416 | 0.218 | 0.332 |
| ASD | 0.492 | 0.137 | 0.067 | 0.359 | 0.273 | 0.462 | 0.323 | 0.37 |
| MDD | 0.278 | 0.231 | 0.206 | 0.082 | 0.042 | 0.304 | 0.438 | 0.47 |
| SCZ | 0.394 | 0.147 | 0.007 | 0.271 | 0.293 | 0.067 | 0.255 | 0.143 |
| EDUyears | **8.89×10^-17^** | **7.94×10^-12^** | **7.52×10^-12^** | **5.81×10^-15^** | **2.75×10^-8^** | 0.329 | 0.027 | 0.025 |
| Accumbens | 0.32 | 0.282 | 0.497 | 0.346 | 0.116 | 0.465 | 0.268 | 0.156 |
| Amygdala | 0.248 | 0.199 | 0.405 | 0.139 | 0.009 | 0.179 | 0.233 | 0.13 |
| Caudate | 0.371 | 0.196 | 0.35 | 0.047 | 0.055 | 0.394 | 0.137 | 0.144 |
| Hippocampus | 0.217 | 0.306 | 0.426 | 0.056 | 0.271 | 0.235 | 0.427 | 0.032 |
| Pallidum | 0.174 | 0.406 | 0.152 | 0.215 | 0.254 | 0.197 | 0.098 | 0.444 |
| Putamen | 0.18 | 0.076 | 0.288 | 0.002 | 0.211 | 0.223 | 0.047 | 0.459 |
| Thalamus | 0.218 | 0.033 | 0.006 | 0.004 | 0.004 | 0.454 | 0.354 | 0.097 |

**Table S11.** Results of the polygenic risk score analyses run in PRSice^1^, comparing the results of our GWAS on DD-related cognitive measures (hereafter called target traits) with twelve different neuroimaging, educational, and neuropyschiatric phenotypes (hereafter called training traits, see main text). Here we report **a)** *Nagelkerke’s* *R^2^* values, representing the proportion of variance explained by each training trait (in rows) for each target trait (in columns), and **b)** the corresponding p-values, at a significance of association (*P_T_*) threshold of 0.05 in the training GWAS. Such results were supported at varying *P_T_* thresholds (see below). Significant *p*-values withstanding Bonferroni correction (*p* < 9.4 × 10^-5^) and the corresponding *R^2^* values are highlighted in bold.

Legend: WRead = word reading; WSpell = word spelling; NWRead = nonword reading; PA = Phoneme Awareness; DigSpan = digit span; RANdig/RANlet/RANpic = Rapid Automatized Naming of digits/letters/pictures; ADHD = Attention Deficit Hyperactivity Disorder^2^; ASD = Autism Spectrum Disorder^3^; MDD = Major Depressive Disorder^4^; SCZ = Schizophrenia^5^; EDUyears = years of education completed (educational attainment)^6^.

c)

| Trait | WRead | WSpell | NWRead | PA | DigSpan | RANdig | RANlet | RANpic |
| --- | --- | --- | --- | --- | --- | --- | --- | --- |
| ADHD | -0.051 | -0.068 | -0.045 | -0.04 | -0.044 | 0.027 | 0.002 | 0.023 |
| ASD | 0.003 | 0.006 | 0.005 | -0.032 | -0.005 | 0.012 | 0.026 | 0.009 |
| MDD | -0.011 | -0.002 | 0.001 | 0.006 | 0.02 | 0.006 | -0.001 | -0.01 |
| SCZ | -0.016 | -0.006 | 0.012 | -0.001 | 0.007 | 0.014 | 0.004 | 0.004 |
| EDUyears | 0.072 | 0.088 | 0.062 | 0.078 | 0.022 | 0.008 | 0.01 | 0.018 |
| Accumbens | -0.03 | -0.017 | -0.036 | -0.021 | 0.015 | -0.004 | -0.027 | 0.006 |
| Amygdala | 0.019 | 0.019 | 0.01 | 0.019 | 0.043 | 0.014 | 0.014 | 0.037 |
| Caudate | -0.026 | -0.0004 | -0.014 | 0.009 | -0.005 | -0.015 | -0.009 | -0.016 |
| Hippocampus | -0.005 | -0.006 | -0.02 | 0.005 | -0.004 | -0.009 | -0.004 | 0.012 |
| Pallidum | -0.002 | -0.001 | 0.001 | 0.002 | -0.029 | -0.008 | -0.007 | 0.021 |
| Putamen | -0.019 | -0.008 | -0.02 | 0.01 | -0.01 | 0.007 | -0.005 | 0.005 |
| Thalamus | -0.036 | -0.025 | -0.028 | 0.001 | -0.01 | -0.03 | -0.032 | -0.018 |

**Table S11c.** Correlation (*Pearson’s R*) coefficient of genetic association β values (r_β_) between the DD-related traits tested in the GWAS (in columns) and twelve different neuroimaging, educational, and neuropyschiatric phenotypes (in rows). These were computed for all the SNPs showing association *p*-values < 0.05 in the training GWAS.

1. ADHD vs. WRead


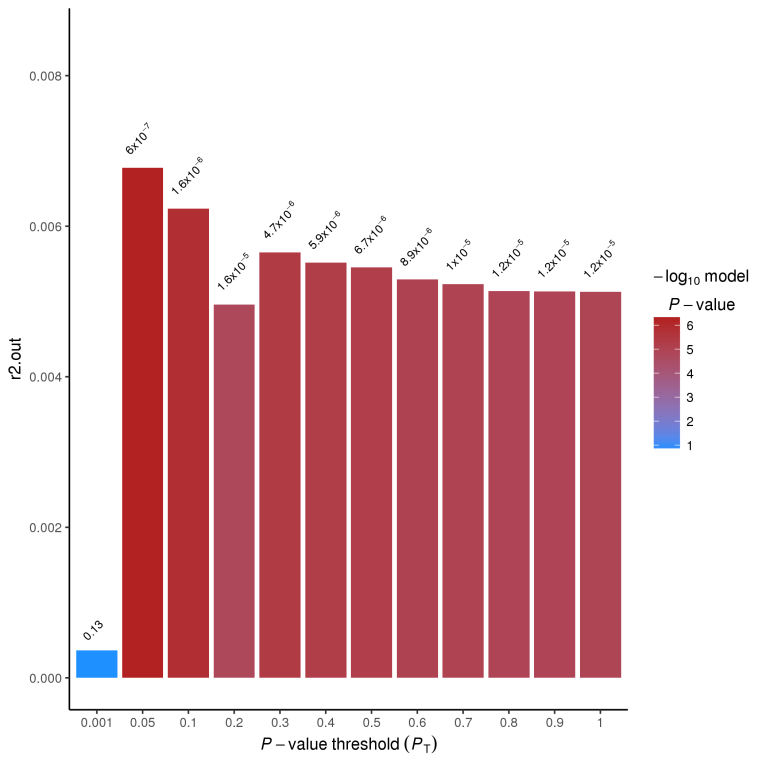


1. ADHD vs. WSpell


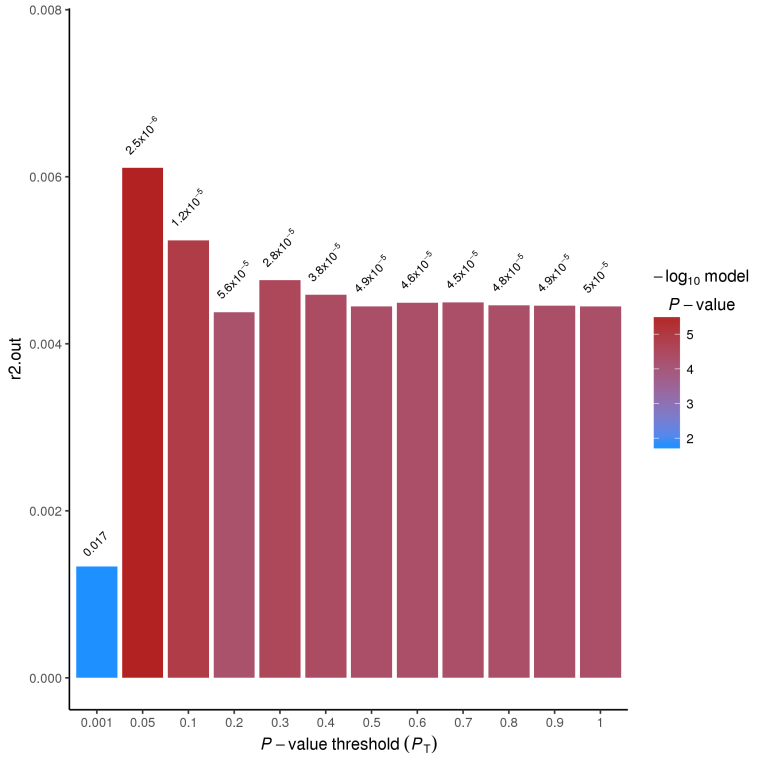


1. ADHD vs. NWRead


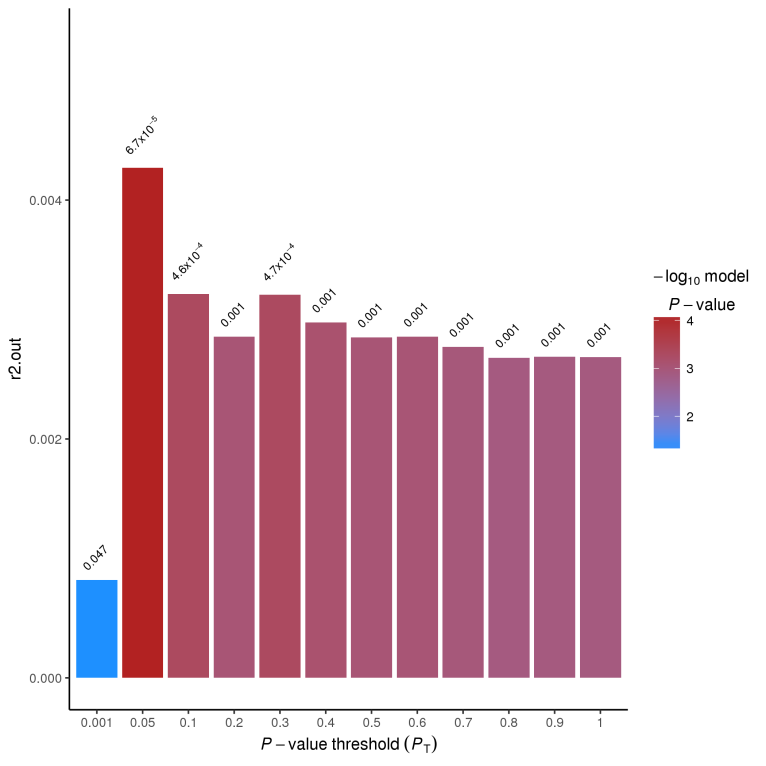


1. EDUyears vs. WRead


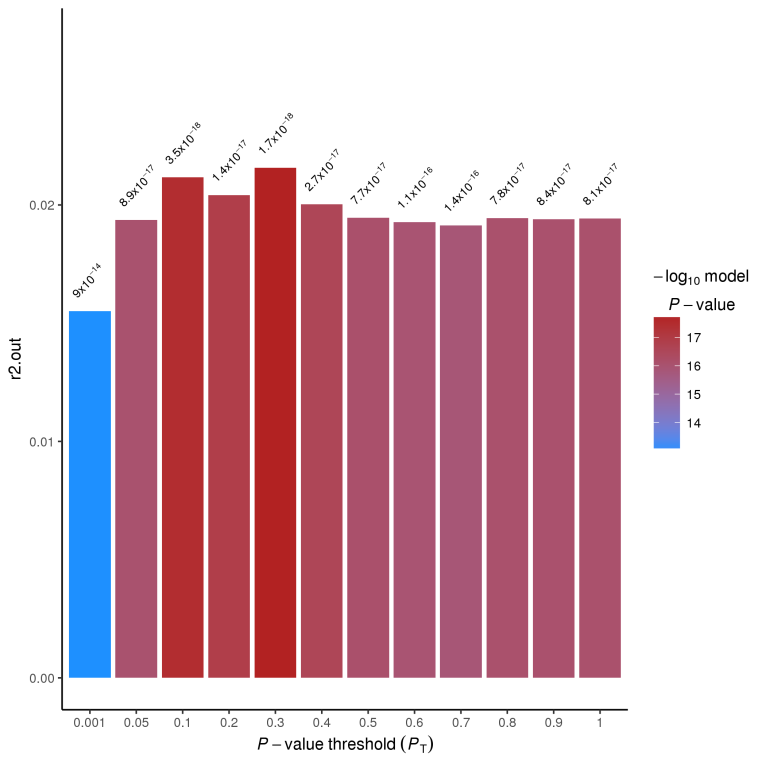


1. EDUyears vs. WSpell


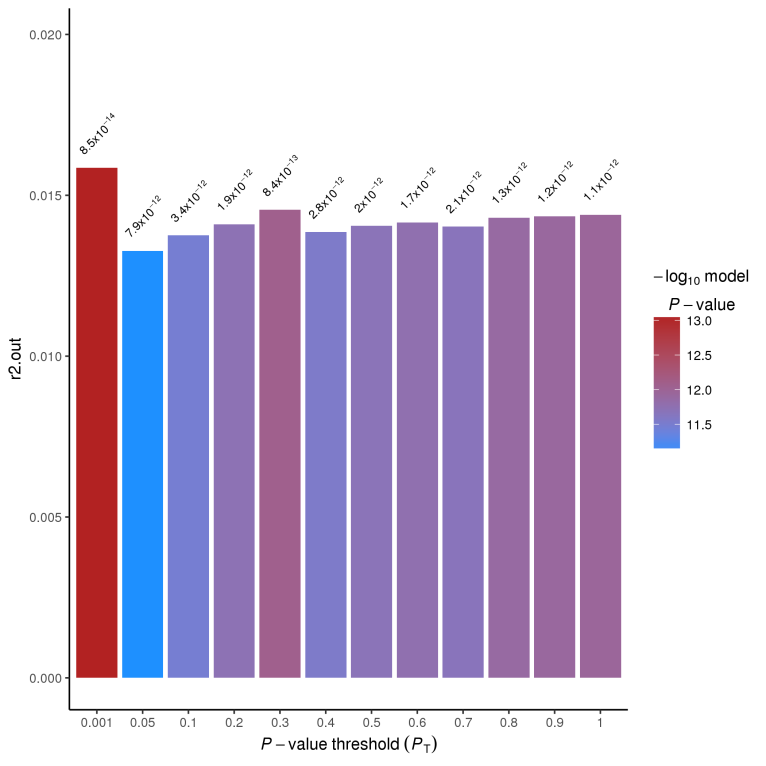


1. EDUyears vs. NWRead


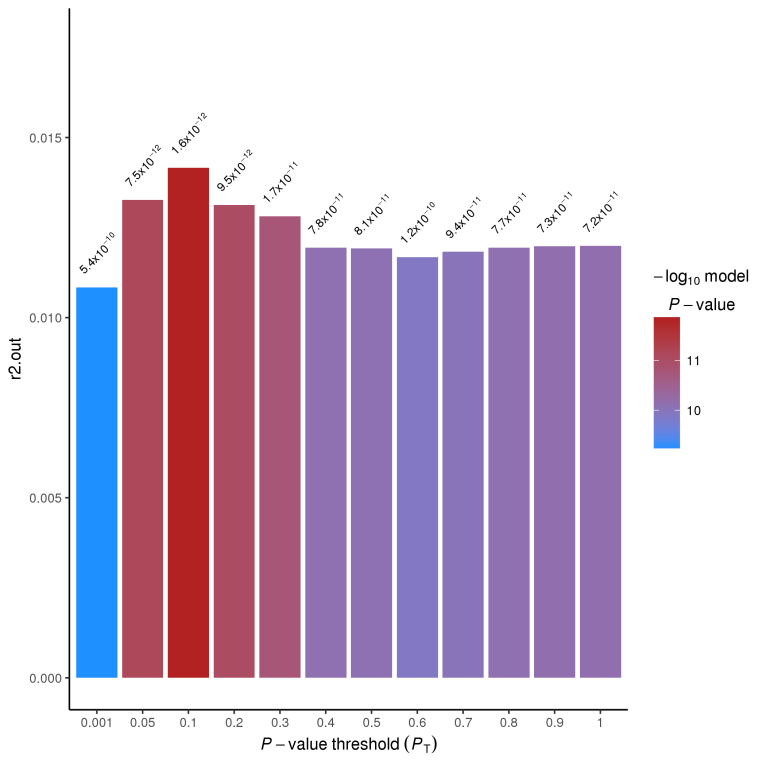


1. EDUyears vs. PA


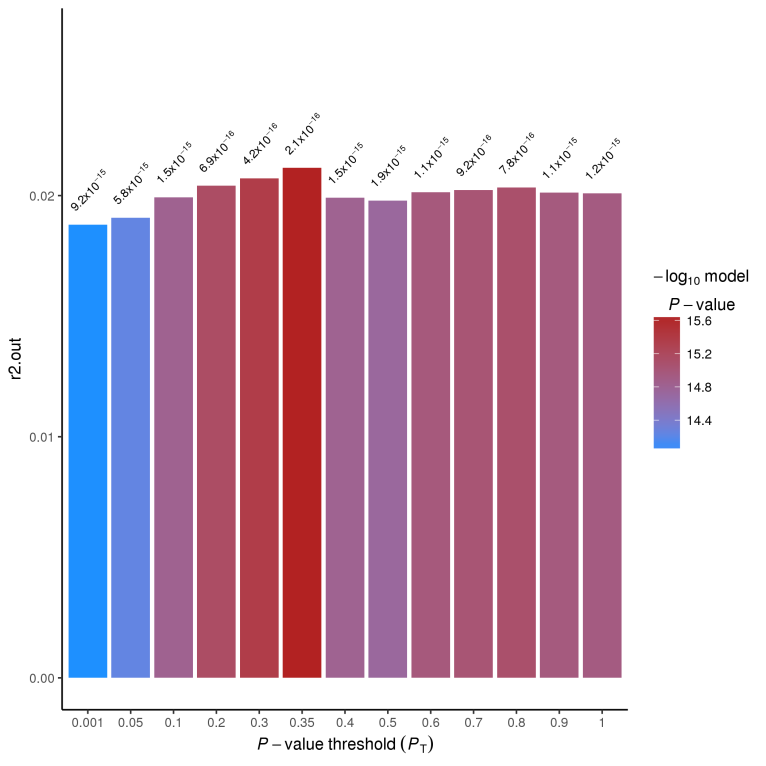


1. EDUyears vs. DigSpan


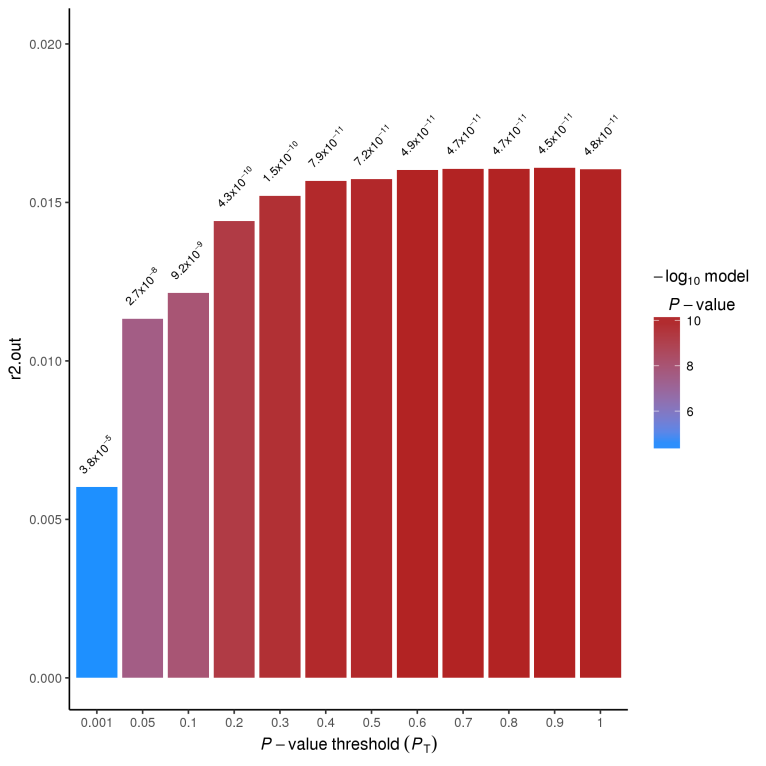


**Figure S11.** Proportion of variance (*Nagelkerke’s R^2^*) explained by the training traits ADHD and EDUyears in some of the target traits tested in the present GWAS, at varying *P_T_* thresholds (0.001, 0.05, 0.1, 0.2, 0.3, 0.4, 0.5, 0.6, 0.7, 0.8, 0.9, 1). On top of each column the relevant *p*-value is reported.

Here we report only cross-trait comparisons which were significant at *P_T_* = 0.05, namely those of ADHD vs. **a)** WRead; **b)** WSpell; **c)** NWRead; and of EDUyears vs. **d)** WRead; **e)** WSpell; **f)** NWRead; **g)** PA, and **h)** DigSpan.

**References**

1 Euesden J, Lewis CM, O’Reilly PF. PRSice: Polygenic Risk Score software. *Bioinformatics* 2015; **31**: 1466–1468.

2 Demontis D, Walters RK, Martin J, Mattheisen M, Als TD, Agerbo E *et al.* Discovery of the first genome-wide significant risk loci for attention deficit/hyperactivity disorder. *Nat Genet* 2018; : 1.

3 Access O. Meta-analysis of GWAS of over 16,000 individuals with autism spectrum disorder highlights a novel locus at 10q24.32 and a significant overlap with schizophrenia. *Mol Autism* 2017; **8**: 21.

4 Sullivan PF, Daly MJ, Ripke S, Lewis CM, Lin DY, Wray NR *et al.* A mega-Analysis of genome-wide association studies for major depressive disorder. *Mol Psychiatry* 2013; **18**: 497–511.

5 Ripke S, Neale BM, Corvin A, Walters JTR, Farh KH, Holmans PA *et al.* Biological insights from 108 schizophrenia-associated genetic loci. *Nature* 2014; **511**: 421–427.

6 Okbay A, Beauchamp JP, Fontana MA, Lee JJ, Pers TH, Rietveld CA *et al.* Genome-wide association study identifies 74 loci associated with educational attainment. *Nature* 2016; **533**: 539–542.

**URLs**

<http://prsice.info/>

<https://www.thessgac.org/data>

<http://www.med.unc.edu/pgc/results-and-downloads>

<http://enigma.ini.usc.edu/research/download-enigma-gwas-results/>
